# Spatial and socio-economic correlates of effective contraception among women seeking post-abortion care in healthcare facilities in Kenya

**DOI:** 10.1371/journal.pone.0214049

**Published:** 2019-03-27

**Authors:** Michael M. Mutua, Thomas N. O. Achia, Lenore Manderson, Eustasius Musenge

**Affiliations:** 1 African Population and Health Research Center (APHRC), Nairobi, Kenya; 2 School of Public Health, University of the Witwatersrand, Johannesburg, South Africa; 3 Centers for Disease Control and Prevention, Nairobi, Kenya; 4 Institute at Brown for Environment & Society (IBES), Brown University, Providence, Rhode Island, United States of America; Conrad, UNITED STATES

## Abstract

**Introduction:**

Information, counseling, availability of contraceptives, and their adoption by post-abortion care (PAC) patients are central to the quality of PAC in healthcare facilities. Effective contraceptive adoption by these patients reduces the risks of unintended pregnancy and repeat abortion.

**Methods:**

This study uses data from the Incidence and Magnitude of Unsafe Abortion Study of 2012 to assess the level and determinants of highly effective contraception among patients treated with complications from an unsafe abortion in healthcare facilities in Kenya. Highly effective contraception was defined as any method adopted by a PAC patient that reduces pregnancy rate by over 99%.

**Results:**

Generally, contraceptive counseling was high among all PAC patients (90%). However, only 54% of them received a modern family planning method—45% a short-acting method and 9% a long-acting and permanent method. Adoption of highly effective contraception was determined by patient’s previous exposure to unintended pregnancies, induced abortion and modern family planning (FP). Facility level factors associated with the uptake of highly effective contraceptives included: facility ownership, availability of evacuation procedure room, whether the facility had a specialized obstetric-gynecologist, a facility that also had maternity services and the number of FP methods available for PAC patients.

**Discussion and conclusion:**

For better adoption of highly effective FP, counseling of PAC patients requires an understanding of the patient’s past experience with contraception and their future fertility intentions and desires in order to meet their reproductive needs more specifically. Family planning integration with PAC can increase contraceptive uptake and improve the reproductive health of post-abortion care patients.

## Introduction

Globally, about 22 million unsafe abortions occur annually, 21 million in low and middle income countries. The World Health Organization (WHO) estimates that 47,000 cases of maternal deaths are due to unsafe abortion, with only 0.2% of these in high-income countries [1]. Over 62% of these deaths occur in Africa, primarily in eastern Africa [2].

Women who have had an abortion may resume sexual activity soon after abortion, around the same time as ovulation resumption. Post-abortion contraception is therefore a necessary intervention to reduce the risk of unintended pregnancies. In consequence, the timing of menses after resumption of ovulation provides a further justification for providing post-abortion contraception with post-abortion care [3,4] to avert further unwanted pregnancies [5] and reduce abortions and abortion-related deaths.

Although post-abortion contraception is a component of all post-abortion care (PAC) models, service providers have focused on the treatment of complications and family planning and counseling have continually been overlooked [4]. The provision of contraception in consultation with a trained service provider increases the rate of use of family planning (FP) in the first six months post-abortion [5], consistent with WHO recommendations for a pregnancy after an abortion or miscarriage [6]. This duration of adoption of a family planning method is shorter when abortion or post-abortion treatment involves a medical abortion with Mifepristone and Misoprostol [3]. This points to the necessity of integrating FP with safe legal abortion and post-abortion care services to address missed opportunities and to reduce the risks of repeat unsafe abortions [7].

In a study conducted on contraceptive knowledge and use among women treated for unsafe abortions in Accra, Ghana, the majority of women seeking care had little knowledge of contraceptive methods. This low knowledge contributed to low uptake of contraception after securing treatment for complications resulting from unsafe abortions [8]. In another cross-country study in India, Pakistan and Bangladesh, better exposure to information was linked to higher contraceptive use among patients [9]. In addition, women seeking multiple abortions are more likely to choose and have used short-acting family planning methods [10]. In similar low-income and high-risk settings, choosing a more effective method was mostly associated with a history of previous abortions and number of living children, a proxy for fertility intentions [11]. At the population level, when fertility is unchanged, increased contraception use and use of more effective methods have been demonstrated to reduce risks of induced abortion. More specifically, the use of intrauterine contraceptive devices (IUCDs) after abortion significantly reduces the risks of repeat abortion. [12].

In Kenya, despite recent increases in contraceptive prevalence, the acceptance of modern methods of family planning remains below 40% [13] for reasons which include fear of side effects, lack of social support, myths and misconceptions, religious beliefs, and the costs of either the method or consultation with provider [13,14]. In areas such as western Kenya, these barriers also include missed opportunities at health facilities, especially for postpartum women [14]. Effective post-abortion contraceptive counseling could, therefore, play a significant role in demystifying contraception and increasing uptake.

The relationship between effective contraceptive counseling and adoption of long-acting contraceptive methods has been shown to vary [15,16]. However, through effective contraception counseling, patients treated with post-abortion complications are more likely to adopt an effective contraceptive method on, before or soon after discharge from the facility [7], even in environments with limited resources that could otherwise facilitate lower costs of care and help to deliver patient-centered care [17–19]. For this to happen, PAC services need to be comprehensive and include the provision of effective contraceptives and the involvement of partners, since male involvement increases the rate of acceptance, especially of long-acting methods, by PAC patients [19]. Increasing acceptance and uptake of effective contraceptive methods through better counseling and male involvement has been demonstrated to hold both in environments of legal abortion and in more restrictive abortion environments [18].

Drawing on Marston and Cleland [12] and Keen and colleagues [11], in this article, we seek to investigate levels and types of post-abortion contraception for women seeking PAC in healthcare facilities in Kenya. We examine the determinants of highly effective post-abortion contraception among PAC patients; describe their contraceptive uptake after treatment; assess the health facility’s preparedness to offer post-abortion contraception; and analyse the nature of barriers to service and method availability.

## Methods

### Study setting

The data are drawn from the Incidence and Magnitude of Unsafe Abortion (IMUA) study conducted in 2012 in Kenya, targeting a nationally representative sample of 350 facilities. The Ministry of Health in Kenya classifies public and private healthcare facilities providing preventive and curative services into six categories based on their operational capacity. These classifications are community health services (Level I); primary care facilities (Level II and III) comprised of dispensaries, clinics, health centers and maternity homes; county referral health facilities (Levels IV and V), comprised of district/county hospitals, sub-district/sub-county and provincial hospitals; and Level VI (comprised of national referral and teaching hospitals) [20]. As at January 31, 2012, when sampling was undertaken, there were a total of 2,838 facilities classified as between Level II (the lowest level of healthcare facilities) and Level IV (the highest referral level).

### Sampling

According to the IMUA Study whose data is used in this analysis, the sample of 350 facilities allowed for detection of a 10% difference in the severity of complications. Sampling fractions ps = 0.1233 (350 out of 2,828 facilities), together with survey response rate pr = 94% (328 out of 350 facilities), were used to compute sampling weights used in this analysis sw = 1/ (ps*pr) = 8.6279. These sampling weights were then normalized and applied differentially for all facilities in the same strata, according to facility level and geographic location. The study collected data from 328 facilities (94 in Level II, 124 in Level III, 94 in Level IV, 14 in Level V and 2 in Level VI); 22 of the 350 sampled facilities did not participate in the study. The sample was stratified by both facility level and region.

### Data

Data were collected from all participating facilities and all PAC patients treated in these facilities over a 30 day period in each facility. Data collection was conducted between April and August 2012. Field interviewers collected facility information by interviewing facility managers or senior staff informed about PAC service delivery in these facilities. Data collected included facility’s infrastructure for PAC, staff training on PAC, number of cases the facilities managed in an average/typical month, common practices of services offered to PAC patients, and information on how these facilities cope with demands for PAC services. One or two PAC service providers in each facility were identified and trained to collect data on all PAC patients who presented in these participating facilities. These service providers collected data from their PAC patients using fully structured questionnaires on women’s socio-economic background characteristics, fertility experiences and planning, contraceptive use, and abortion histories, as well as data on the step-by-step management of these patients until they were discharged from their facility. By the end of the data collection period, the study had gathered patient data for 2,568 patients who sought PAC over the 30 days at 281 of the 328 participating facilities, with the other 47 facilities either not reporting any PAC cases during the observation period, or only reporting cases of patients that sought termination of pregnancy. This study focuses on post-abortion care patients only in order to assess the management of patients presenting with complications resulting unsafe abortions. Consequently, an additional 466 patients (15.4%) who sought termination of pregnancies were excluded from this analysis.

### Variables

The primary outcome variable, contraceptive use, was based on responses to three questions on whether a patient treated for PAC received a modern contraceptive method: 1) whether the patient was counseled for FP before leaving the health facility: 2) whether the patient was given a modern method of FP before leaving the health facility; and 3) type of contraceptive method the patient received. The outcome variable is defined as a patient who was counseled for and given a highly effective method of FP. All other patients were classified into those who received a “less effective” method or no method at all. According to Hatcher’s classification of contraceptives based on use or actual effectiveness rather than theoretical effectiveness [21], highly effective contraceptive methods include methods that, irrespective of user’s compliance or consistency, reduce pregnancy risk by 99%. These include only long-acting and reversible contraceptives (LARCs) or long-acting and permanent methods (LAPM) that guarantee high efficacy for pregnancy prevention in typical use scenario. These include contraceptive implants, IUCD, and female sterilization [11,22]. These methods all address user challenges related to human error, distance to health facility, and inconsistent use. This outcome variable is considered ordinal, measuring a latent continuum of the efficacy of a contraceptive method in preventing unintended or unwanted pregnancy. A patient discharged on a highly effective method is therefore considered at the lowest risk of repeat abortion, while one who received a less effective method is at moderate risk. A patient who did not receive any method was considered to be at the highest risk of unintended pregnancy and hence at risk of repeat abortion. Since data collection was completed by service providers, who were integral in determining whether a patient received a highly effective contraceptive method or not, we measured certain provider and facility characteristics that we hypothesized influenced this outcome of interest. These include facility level, ownership, whether a facility had a gynecological or FP unit, number of staff trained to offer PAC, and whether or not such trained providers were always available to attend to PAC patients. We also considered demographic information of the main service provider at the facility: gender, age, and duration of service in the facility.

At the county-level, data were collected on three main categories. Proximate determinants of contraceptive use measures of health care seeking behavior and socio-economic determinants of contraceptive use were both considered. Among these variables were measures such as county level of unmet need for contraception, fertility intention, and exposure to contraceptive information. We also included a measure of women’s agency in decision-making for contraceptive use and community-level measures of access to healthcare, such as the percent of deliveries conducted by a skilled provider and the number of pregnant women in the past five years who received a tetanus toxoid injection. Finally, to account for any socio-economic variability among the counties, a measure of county level religious affiliation was considered, by including the percentage of Christians (the main religion in the country), a relative self-reported measure of food security, household size, and a measure of county-level of investment in health through county budgetary allocation to health. These measures were first tested for serial correlation and then selected based on the level of correlation between each other, using a 0.7 correlation coefficient cutoff with any other variable within the above three subcategories.

### Survey ethics considerations

The parent study on which this analysis is based was reviewed and approved by the Ethical Review Boards of the Kenya Medical Research Institute, the University of Nairobi/ Kenyatta National Hospital, Moi University Teaching and Referral Hospital, Kenya, and Aga Khan University, Kenya.

In addition to these ethics reviews, the Ministries of Public Health and Sanitation and Ministry of Medical Services (now both under Ministry of Health) in Kenya and the Institutional Review Board of the Guttmacher Institute also reviewed and approved the study. During the collection of field data, verbal consent was obtained from all women presenting for care prior to data collection, while written consents were obtained from all facility managers who were interviewed for the facility component. As per the requirements of the ethics boards, all verbal consents were recorded in the data collection tool by reading the consent statement and allowing the respondent to confirm their consent to participate. The interviewer then signed on the data collection tool that consent had been obtained. In this study, minors were considered as emancipated by virtue of having been pregnant, as per the Kenya adolescent research guidelines. Data collectors and service providers were all taken through the research ethics in human participant surveys, with attention to the key elements of respect, beneficence, and justice in research on human subjects. All participants were assured of strict confidentiality.

### Data management and analysis

All data were obtained for analysis in the form of Stata datasets. Initial consistency checks and data re-coding were conducted in Stata 13 [23]. All new variables were generated, tested for consistency with original variable, and stored as new variables while maintaining the original variables. Descriptive statistics of the facility and service provider characteristics were computed and are presented in Table 1 (below). All analysis conducted to produce descriptive statistics were weighted using the survey weights in order to account for the survey design.

**Table 1:** Description of healthcare facilities according to facility and service provider characteristics, 2012.

	% of sample/ Mean	Number of facilities/Main service providers
Health facility Characteristics		
***Facility level***		
Level 2	47.6	71
Level 3	36.0	105
Level 4	15.6	89
Level 5	0.7	14
Level 6	0.1	2
***Region***		
Central & Nairobi	14.1	65
Coast & N. Eastern	16.6	48
Eastern	10.7	50
Nyanza & Western	30.0	63
Rift valley	28.5	55
***Ownership***		
Public	68.5	176
Private for Profit	16.5	52
Private not for profit	15.1	53
***Facility with FP or Gynecology unit***		
No	18.0	43
Yes	82.0	238
***PAC-trained staff always available***		
Not always available	57.6	137
Always available	42.4	144
***Average No*. *of staff trained to offer PAC***	Mean = 2.13	281
***Service Provider Characteristics***		
***Cadre of main PAC service provider***	**%**	**N**
Doctor	26.9	87
Nurse	68.2	187
Other	4.9	7
***Age of main PAC provider***		
<30 yrs	27.7	70
30–39 yrs	33.3	94
>40 yrs	39.1	117
***Gender of main PAC provider***		
Male	52.3	138
Female	45.7	141
Missing	2.0	2
***Average Duration of service in current facility***	Mean = 4.99	281
**Total**	**100.0**	**281**

Statistical modeling was first conducted in Stata 15 using bivariate and multivariable mixed-effects ordered logit models in the Bayesian framework using Stata and OpenBUGS software [24]. Bayesian statistics have in the recent past demonstrated critical advantages over maximum likelihood estimation procedures [25]. This includes Bayesian ability to assign prior information to data, without overreliance on available data alone.

The outcome variable is ordinal, and use of ordered logit models extends the logistic regression models to allow for modeling data which assumes an underlying sequentially incremental nature of a rather categorical outcome. We further considered the use of mixed effects models because theoretically, women treated in one facility (women nested in facilities), as well as those treated in facilities that are in the same county (facilities nested in counties) have more similar treatment experiences than women treated elsewhere. In this analysis, the second level of nesting of facilities in counties was ignored because there were too few facilities in some counties to provide any meaningful structural variability at that level. The final models therefore considered only two levels of women nested in counties, while assuming constant variability between and within facilities.

To account for the multilevel nature of these data, we introduced unstructured random effects in order to capture unobserved non-spatial heterogeneity [25]. Subsequently, we fitted an ordered logit model with structured random effects, using a conditional autoregressive (CAR) model to account for the spatial dependency between counties that are close to each other than those that are far apart [26,27]. Finally, we fitted a convoluted model, combining both the above unstructured and structured spatial random effects in our final model, a special flexibility provided within the framework of Bayesian estimation models [28]. All covariates were first tested for inclusion at 85.0% credibility interval, where initial bivariate models were fitted and all variables that were significant at this more relaxed credibility interval (85%) were considered for inclusion in the final model. Only facility ownership (public/private) failed to meet this inclusion criterion, but we still included this due to its contextual importance to this study.

Within each model, deviance information criterion (DIC) was used to compare models based on goodness of fit, with models with the lowest DIC value having the best fit. Posterior estimates from these models were plotted on county maps in ArcGIS software [29] to visualize the geographical or regional variability in the adoption of highly effective contraceptive methods by PAC patients in health care facilities.

## Results

The survey sample consisted mainly of Level II and Level III facilities (83.6% of the sample). Although most facilities were publicly owned and managed, 105 facilities were owned either by private individuals or non-governmental organizations.

On average, there are about two service providers in each facility with training in PAC, with only 42.4% of the facilities reporting that these trained providers were always available to offer PAC whenever the facilities were operational. For the service providers who were interviewed, 68.2% were nurses and 27.7% were doctors involved in PAC provision in their facilities. The majority was aged 30 years and above (72.4%), with slightly more men (52.3%), with an average of five years of continuous PAC service delivery in the sampled facilities.

Although these facilities offered PAC, 18.0% did not have gynecology or FP units. This was particularly the case in Level II (22.7%) and for-profit (26.3%) facilities in central and Nairobi regions (30.0%), although these differences were not statistically significant. On average, there were two trained PAC providers per facility, with major differences according to facility level. Level II facilities on average had less than one provider, while Level VI had more than 35 providers trained to offer PAC. Nyanza, Western and Rift valley regions had the lowest number of providers with in-service training on PAC compared to other regions. For-profit facilities reported a higher number of trained providers, but providers were not always available for PAC service provision, and availability varied by facility level and region. Level II facilities reported the lowest rates of availability (33.6%), then Level IV (47.1%) and Level III (51.0%). Level V and VI had the highest rates of availability (79.6% and 100% respectively), as shown in Table 2.

**Table 2:** Description of healthcare facilities by contraceptive characteristics according to level, region and ownership, Kenya 2012.

	Overall	**Facility Level**						***Region***						***Ownership***				
	% and Mean	**Level 2**	**Level 3**	**Level 4**	**Level 5**	**Level 6**	*Chi*^*2*^; Design-based F*; p value	Central & Nairobi	Coast & N. Eastern	Eastern	Nyanza & Western	Rift valley	Chi2; Design-based F*; p value	Public	Private for Profit	Private not for profit	Chi2; Design-based F*; p value	Number of facilities
***Facility with FP or Gynaecology unit***	82.0	77.3	85.3	88.0	100.0	100.0	4.28; 1.79; p>0.1	70.0	82.7	88.1	83.7	83.6	4.96; 0.79; p>0.1	84.6	79.1	73.7	3.12; 0.89; p>0.1	238
***Average No*. *of staff trained to offer PAC***^***¥***^	2.129 (1.853–2.404)	0.982 (0.706,1.259)	2.101 (1.711,2.491)	4.954 (3.794,6.114)	14.716 (8.04,21.392)	35 (25.157,44.843)	F = 823.95; Prob > F = 0.0000	2.853 (2.13,3.577)	2.513 (1.679,3.348)	2.954 (1.678,4.231)	1.779 (1.27,2.288)	1.604 (1.093,2.115)	F = 70.30; Prob > F = 0.0000	1.915 (1.598,2.231)	2.944 (2.113,3.776)	2.21 (1.358,3.061)	F = 133.37; p< = 0.001	281
*PAC-trained staff always available*	42.4	33.6	51.0	47.1	79.6	100.0	9.10; 3.39; p<0.05	65.2	47.2	50.1	31.0	37.3	14.92; 2.70; p<0.05	40.8	53.7	36.9	3.13; 1.05; p>0.1	144
***FP availability***																		
Offers effective FP and non-effective methods	70.0	54.0	85.2	82.7	93.8	50.0	34.99; 5.88; p<0.001	92.0	59.9	64.1	75.0	61.9	18.62;1.72; p>0.1	71.1	77.3	57.2	35.92; 5.28; p<0.001	218
Offers non-effective methods only	27.9	44.1	13.2	12.9	6.2	50.0		8.0	34.7	33.6	25.0	34.6		28.9	22.7	28.6		57
No methods offered	2.1	1.9	1.6	4.4	0.0	0.0		0.0	5.4	2.3	0.0	3.5		0.0	0.0	14.2		6
***To whom FP counselling is offered ALWAYS***																		
Everyone	80.6	75.1	85.9	84.9	87.7	100.0	5.05; 1.95; p>0.1	80.2	99.2	80.8	87.9	62.3	30.41; 6.96; p<0.001	78.5	93.8	76.0	6.27; 2.23; p>0.1	236
Those who have many children	82.6	79.3	85.9	84.9	87.7	100.0	2.00; 0.77; p>0.1	80.2	99.2	80.8	87.9	69.4	20.62; 4.69; p<0.005	81.4	93.8	76.0	5.50; 1.98; p>0.1	238
Those who are married	83.8	79.3	89.0	84.9	87.7	100.0	4.09; 1.62; p>0.1	80.2	99.2	80.8	87.9	73.3	16.24; 3.64; p<0.05	82.2	93.8	79.7	4.24; 1.49; p>0.1	240
Those who are of an older age	80.6	75.1	85.9	84.9	87.7	100.0	5.04; 1.95; p>0.1	80.2	99.2	80.8	87.9	62.3	30.41; 6.96; p<0.001	78.5	93.8	76.0	6.27; 2.23; p>0.1	236
***Topics covered in FP counselling***																		
Instructions on correct use of all methods	64.6	62.4	67.3	63.4	85.8	100.0	1.15; 0.42; p>0.1	74.7	85.3	67.8	51.3	59.9	18.15; 3.45; p<0.05	69.1	68.4	38.2	14.25; 4.62; p<0.05	189
Instructions on available methods	82.9	78.0	88.2	84.4	93.8	100.0	4.53; 1.68; p>0.1	87.9	86.3	86.9	88.6	70.0	12.30; 1.99; p>0.1	85.7	84.3	67.9	7.45; 2.03; p>0.1	242
Instructions on traditional methods only	8.4	10.7	4.9	9.6	6.2	0.0	2.63; 0.99; p>0.1	2.2	12.7	8.5	12.8	3.8	7.43; 1.19; p>0.1	8.4	0.0	18.1	9.24; 2.77; p<0.1	20
Advantages & disadvantages of each	78.3	75.9	79.6	81.6	93.8	100.0	1.18; 0.45; p>0.1	86.9	75.5	71.6	75.5	81.4	3.60; 0.63; p>0.1	77.0	87.5	73.6	3.03; 1.19; p>0.1	226
What to do with method failure or forgetting pills	19.7	22.1	19.0	13.7	32.7	0.0	1.79; 0.663; p>0.1	22.1	32.0	1.2	7.7	31.8	25.99; 4.97; p<0.005	20.1	21.2	16.3	0.38; 0.11; p>0.1	53
Abstinence	8.3	3.9	11.1	14.3	20.4	0.0	6.88; 2.09; p>0.1	7.8	7.3	3.1	16.4	2.1	12.30; 2.84; p<0.05	6.1	1.3	27.1	22.83; 12.05; p<0.001	25
General FP	8	5.1	9.9	12.4	0.0	0.0	3.30; 1.11; p>0.1	9.8	0.8	2.7	9.2	12.3	6.72; 1.65; p>0.1	6.7	7.2	15.1	3.22; 0.97; p>0.1	24
Dangers of abortion	6.7	6.3	9.2	1.5	24.7	0.0	3.96; 1.30; p>0.1	3.3	0.0	6.1	7.7	11.9	7.50; 1.27; p>0.1	7.5	2.3	8.0	1.81; 0.74; p>0.1	16
Need for follow up visits	4.5	8.9	0.4	1.4	0.0	50.0	12.04; 7.60; p<0.001	0.0	0.3	0.0	0.1	16.4	33.73; 9.96; p<0.001	6.1	0.3	2.2	3.54; 4.38; p<0.05	9
Duo-approach for FP/STI/HIV	9.5	8.6	11.1	8.7	0.0	0.0	0.69; 0.24; p>0.1	5.5	0.8	0.0	20.7	8.2	20.74; 5.14; p<0.005	12.6	0.0	5.9	7.63; 2.59; p<0.1	21
Total	100	100.0	100.0	100.0	100.0	100.0		100.0	100.0	100.0	100.0	100.0		100.0	100.0	100.0		281

^¥^ Pr(|T| > |t|) based on student t-test for means.

* Rao and Scott second-order corrected Pearson statistic.

Almost two-thirds of all facilities reported that they stocked and offered highly effective family planning methods to PAC patients. An additional 27.9% of facilities offered only short-acting (less effective) methods to PAC patients, with a small proportion of facilities offering no FP methods. Level III, IV and V facilities had the highest rate of reported availability of highly effective FP methods for PAC patients. Nairobi &Central as well as Nyanza & Western regions reported the highest rates (92.0% and 75.0% respectively), with the highest proportion of facilities offering only less effective methods reported in Coast and North-Eastern, Eastern and Rift Valley regions. There was some evidence of facility-based barriers to FP access among PAC patients: about 80.6% of facilities reported always offering FP to all PAC patients, but only 62.3% reported doing so in the Rift Valley.

On topics covered when counseling PAC patients for FP, the majority of the facilities (82.6%) reported covering instructions on available methods of FP, while only 19.7% of facilities reported coverage of patient remedy in case of method failures.

Among all 2,568 patients managed for post-abortion complication in health facilities, 91.8% (2,358) reported receiving counseling on family planning. However, only about 55.5% (1,424) received a modern method of family planning, while, 46.4% received short-acting methods, among them pills (18.2%), injectable contraceptives (22.5%), and condoms (6.6%). Further, only 9.1% (233) received a highly effective method of family planning. As shown in Table 3, 45.3% of all patients did not receive any contraceptive method after treatment for complications from unsafe abortion.

**Table 3:** Description of patients treated for PAC in healthcare facilities according to type of contraceptive method received, by facility characteristics, 2012.

	% No method	% Less-effective	% Highly effective	% Total	Chi^2^; Design-based F*; p value	Number of women
Health facility Characteristics						
Overall	45.3	45.6	9.1	100		2,568
***Facility level***						
Level 2	38.9	51.2	10.0	100.0	48.7; 1.06; p>0.1	231
Level 3	49.1	40.5	10.4	100.0		796
Level 4	49.2	42.6	8.2	100.0		835
Level 5	39.8	58.5	1.7	100.0		530
Level 6	31.3	67.0	1.7	100.0		176
***Region***						
Central & Nairobi	46.1	41.4	12.5	100.0	103.0; 2.26; p<0.05	839
Coast & N. Eastern	50.3	44.9	4.8	100.0		337
Eastern	64.0	32.0	3.9	100.0		363
Nyanza & Western	33.4	55.3	11.3	100.0		505
Rift valley	50.6	42.9	6.6	100.0		524
***Ownership***						
Public	42.0	50.6	7.3	100.0	51.0; 1.96; p>0.1	1,683
Private for Profit	51.6	35.2	13.2	100.0		462
Private not for profit	49.8	40.0	10.2	100.0		423
***Facility with FP or Gynae unit***						
No Facilities	54.5	36.0	9.4	100.0	15.2; 0.92; p>0.1	219
Facilities	43.9	47.1	9.0	100.0		2,349
***PAC-trained staff always available***						
Not always available	44.8	47.1	8.1	100.0	3.6; 0.27; p>0.1	907
Always available	45.8	44.3	9.9	100.0		1,661
Average No. of staff trained to offer PAC^¥^	4.42 (3.13–5.72)	4.47 (3.14–5.79)	3.06 (1.84–4.28)	4.32 (3.37–5.27)		2,568
**No. of women**	**1,144**	**1,191**	**233**	**2,568**		**2,568**

^¥^ Pr(|T| > |t|) based on student t-test for means.

* Rao and Scott second-order corrected Pearson statistic

The use of highly effective contraception was most common in Level II and III facilities (10.0% and 10.4% respectively), followed closely by level IV facilities (8.2%). Slightly under two percent of PAC patients treated at level V and VI facilities received a highly effective contraceptive method. Use of highly effective contraceptive methods was higher in Nairobi/Central and Nyanza/ Western regions (12.5% and 11.3% respectively) than elsewhere. Private for-profit facilities and private not-for-profit facilities had higher rates of highly effective contraception (13.2% and 10.2% respectively).

In order to explore the factors that influenced uptake of highly effective contraception, we fitted multilevel (mixed-effects) model, with two levels of variability; at woman and facility level and county level [30]. The results are presented in four models in Table 4. Model 0 presents results from a bivariate Bayesian mixed-effects ordered logit model of the three-level outcome defined as whether a PAC patient received a highly effective contraceptive method of, a less effective method or whether a patient did not receive any method at all on each patient, facility and county characteristics covariates. Models 1 and 2 present results from an unstructured Bayesian multivariable mixed-effects ordered logit model of the outcome on all covariates, fitted in Stata and BUGs environments respectively. Model 3 was structured, including the county spatial random effects, with conditional autoregressive structure. The final model (Model 4), the results of which are discussed in this article, was a convolution model, with both county non-spatial random effects in model 2 and spatial random effects in model 3 [24]. The model exhibited the lowest DIC value of 3797.0.

**Table 4:** Mixed-effects odds of PAC patients adopting a highly effective contraceptive method in healthcare facilities in Kenya.

		STATA MODEL 0 Bivariate women, facility and county level characteristics	STATA MODEL 1 Unstructured women, facility and county-level characteristics	BUGS MODEL 2: Unstructured Ordered Logit Model	BUGS MODEL 3: Structured Ordered Logit Model	BUGS MODEL 4: Convolution model
		OR (SD) [2.5% - 97.5% Cr. I]	OR (SD) [2.5% - 97.5% Cr. I]	OR (SD) [2.5% - 97.5% Cr. I]	OR (SD) [2.5% - 97.5% Cr. I]	OR (SD) [2.5% - 97.5% Cr. I]
***Patient characteristics***						
Age category: Ref (10-19yrs)	20-29yrs	0.884(0.086)[0.707–1.098]	**0.907(0.010)[0.886–0.932]**	0.868(0.172) [0.622–1.214]	0.868 (0.172) [0.622–1.214]	0.868(0.172) [0.622–1.214]
	30 +yrs	1.242(0.114)[1.013–1.536]	**0.849(0.024)[0.800–0.908]**	0.822(0.188) [0.571–1.186]	0.822 (0.188) [0.571–1.186]	0.822(0.188) [0.571–1.186]
Marital status: Ref (Never married)	Married/Living together	1.042(0.106)[0.835–1.299]	0.984(0.032)[0.924–1.066]	1.192(0.154) [0.881–1.599]	1.192 (0.154) [0.881–1.599]	1.192(0.154) [0.881–1.599]
	Divorced	1.476(0.277)[0.939–2.197]	**1.225(0.054)[1.133–1.371]**	1.249(0.226) [0.796–1.942]	1.249 (0.226) [0.796–1.942]	1.249(0.226) [0.796–1.942]
Education: Ref (No education)	Primary	0.955(0.094)[0.773–1.175]	0.831(0.086)[0.687–1.053]	0.881(0.274) [0.519–1.506]	0.881 (0.274) [0.519–1.506]	0.881(0.274) [0.519–1.506]
	Secondary	0.916(0.086)[0.732–1.114]	0.939(0.086)[0.795–1.186]	0.897(0.289) [0.508–1.558]	0.897 (0.289) [0.508–1.558]	0.897(0.289) [0.508–1.558]
	Post-secondary	1.308(0.166)[0.981–1.743]	1.158(0.089)[0.983–1.385]	1.155(0.324) [0.607–2.135]	1.155 (0.324) [0.607–2.135]	1.155(0.324) [0.607–2.135]
Occupation: Ref (Farmer/unskilled)	Skilled/clerical	1.388(0.142)[1.105–1.733]	**0.915(0.021)[0.87–0.960]**	1.072(0.155) [0.791–1.448]	1.072 (0.155) [0.791–1.448]	1.072(0.155) [0.791–1.448]
	Student	0.814(0.106)[0.602–1.066]	**0.718(0.038)[0.633–0.807]**	0.775(0.209) [0.513–1.168]	0.775 (0.209) [0.513–1.168]	0.775(0.209) [0.513–1.168]
	Unemployed/housewife	0.779(0.081)[0.619–0.985]	**0.793(0.034)[0.714–0.857]**	0.823(0.134) [0.633–1.064]	0.823 (0.134) [0.633–1.064]	0.823(0.134) [0.633–1.064]
Religion: Ref (Roman Catholic)	Other Christian	1.020(0.105)[0.807–1.278]	0.936(0.034)[0.870–1.024]	0.985(0.115) [0.786–1.239]	0.985 (0.115) [0.786–1.239]	0.985(0.115) [0.786–1.239]
	Muslim	0.563(0.127)[0.342–0.917]	**0.599(0.02)[0.559–0.650]**	0.609(0.256) [0.369–1.014]	0.609 (0.256) [0.369–1.014]	0.609(0.256) [0.369–1.014]
	Other religion	2.464(1.113)[0.842–5.718]	**1.745(0.066)[1.612–1.896]**	2.17(0.448) [0.91–5.165]	2.17 (0.448) [0.91–5.165]	2.17(0.448) [0.91–5.165]
Severity level^β^:: Ref (Mild)	Moderate	1.125(0.112)[0.896–1.408]	**1.268(0.048)[1.175–1.373]**	1.029(0.127) [0.801–1.321]	1.029 (0.127) [0.801–1.321]	1.029(0.127) [0.801–1.321]
	Severe	0.840(0.091)[0.654–1.044]	0.975(0.073)[0.814–1.139]	0.777(0.14) [0.587–1.023]	0.777 (0.14) [0.587–1.023]	0.777(0.14) [0.587–1.023]
Gestational age: Ref (First trimester)	Second trimester	0.827(0.083)[0.664–1.050]	**0.861(0.049)[0.759–0.977]**	0.904(0.104) [0.737–1.11]	0.904 (0.104) [0.737–1.11]	0.904(0.104) [0.737–1.11]
Gravidity: Ref (Paragravida) ^§^	Multigravida	1.224(0.109)[1.017–1.501]	**1.213(0.076)[1.049–1.395]**	1.202(0.142) [0.906–1.587]	1.202 (0.142) [0.906–1.587]	1.202(0.142) [0.906–1.587]
	Grand multigravida	1.245(0.151)[0.940–1.656]	**1.266(0.057)[1.143–1.369]**	1.311(0.199) [0.884–1.94]	1.311 (0.199) [0.884–1.94]	1.311(0.199) [0.884–1.94]
Previous abortions: Ref (None)	One or more	2.154(0.420)[1.329–3.212]	**1.950(0.100)[1.712–2.161]**	**1.818(0.209) [1.206–2.748]**	1.818 (0.209) [1.206–2.748]	**1.818(0.209) [1.206–2.748]**
Contraception: Ref (Not using modern)	Using modern	1.898(0.202)[1.503–2.386]	**1.756(0.067)[1.613–1.912]**	**1.637(0.113) [1.312–2.042]**	1.637 (0.113) [1.312–2.042]	**1.637(0.113) [1.312–2.042]**
Pregnancy wantedness: Ref (Wanted then)	Wanted later	1.343(0.143)[1.054–1.700]	**1.358(0.050)[1.252–1.478]**	**1.519(0.131) [1.169–1.961]**	1.519 (0.131) [1.169–1.961]	**1.519(0.131) [1.169–1.961]**
	Did not want	1.285(0.131)[1.004–1.608]	**1.314(0.025)[1.259–1.371]**	**1.605(0.142) [1.22–2.124]**	1.605 (0.142) [1.22–2.124]	**1.605(0.142) [1.22–2.124]**
Referred: Ref (Not referred)	Referred	1.130(0.114)[0.889–1.406]	1.062(0.033)[0.99–1.136]	1.064(0.107) [0.864–1.314]	1.064 (0.107) [0.864–1.314]	1.064(0.107) [0.864–1.314]
***Facility characteristics***						
	Level of facility 4–5 (Ref: Level 2–3)	0.572(0.150)[0.323–0.967]	**0.430(0.023)[0.375–0.466]**	**0.45(0.317) [0.246–0.847]**	0.45 (0.317) [0.246–0.847]	**0.45(0.317) [0.246–0.847]**
	Public (Ref: Private)	1.231(0.359)[0.628–2.188]	**2.784(0.148)[2.428–3.078]**	**2.604(0.295) [1.456–4.591]**	2.604 (0.295) [1.456–4.591]	**2.604(0.295) [1.456–4.591]**
	Facility has evacuation room	2.911(0.440)[2.051–4.111]	**3.340(0.106)[3.106–3.582]**	**2.921(0.161) [2.144–4.035]**	2.921 (0.161) [2.144–4.035]	**2.921(0.161) [2.144–4.035]**
	Ob-gyn (Ref: None)	1.044(0.306)[0.498–1.918]	**1.672(0.039)[1.581–1.75]**	1.125(0.32) [0.608–2.136]	1.125 (0.32) [0.608–2.136]	1.125(0.32) [0.608–2.136]
	Facility has maternity services	0.384(0.159)[0.159–0.783]	**0.613(0.019)[0.577–0.656]**	**0.423(0.364) [0.205–0.869]**	0.423 (0.364) [0.205–0.869]	**0.423(0.364) [0.205–0.869]**
	Burden of PAC to facility (Ref: Not burden)	1.588(0.377)[0.983–2.629]	**1.362(0.095)[1.182–1.595]**	1.456(0.274) [0.839–2.522]	1.456 (0.274) [0.839–2.522]	1.456(0.274) [0.839–2.522]
	No. of PAC trained providers	-	**-**	1.007(0.173) [0.715–1.433]	1.007 (0.173) [0.715–1.433]	1.007(0.173) [0.715–1.433]
	Trained PAC SP always available (Ref: Not)	1.375(0.462)[0.687–2.666]	1.052(0.053)[0.940–1.164]	1.039(0.307) [0.571–1.912]	1.039 (0.307) [0.571–1.912]	1.039(0.307) [0.571–1.912]
	Number of FP methods available	1.247(0.065)[1.098–1.379]	**1.130(0.020)[1.086–1.175]**	**1.191(0.071) [1.041–1.378]**	1.191 (0.071) [1.041–1.378]	**1.191(0.071) [1.041–1.378]**
	Quality of PAC at facility	1.000(0.001)[0.998–1.001]	0.998(0.001)[0.996–1.000]	0.999(0.001) [0.997–1.001]	0.999 (0.001) [0.997–1.001]	0.999(0.001) [0.997–1.001]
***County characteristics***						
******	Level of unmet need for FP	1.005(0.023)[0.970–1.063]	1.004(0.018)[0.965–1.046]	1.007(0.026) [0.959–1.06]	1.007 (0.026) [0.959–1.06]	1.007(0.026) [0.959–1.06]
	% of exposure to FP on radio	1.006(0.005)[0.996–1.016]	1.005(0.004)[0.996–1.014]	1.007(0.005) [0.996–1.017]	1.007 (0.005) [0.996–1.017]	1.007(0.005) [0.996–1.017]
	% of ANC attended by skilled SP	1.027(0.009)[1.010–1.047]	0.981(0.014)[0.950–1.011]	**0.962(0.012) [0.943–0.993]**	0.962 (0.012) [0.943–0.993]	**0.962(0.012) [0.943–0.993]**
	% tetanus vaccine	1.016(0.009)[1.001–1.039]	1.007(0.011)[0.982–1.032]	1.015(0.018) [0.984–1.048]	1.015 (0.018) [0.984–1.048]	1.015(0.018) [0.984–1.048]
	Natural log of county budget allocation to health	1.136(0.041)[1.050–1.218]	1.050(0.057)[0.958–1.190]	1.114(0.077) [0.939–1.237]	1.114 (0.077) [0.939–1.237]	1.114(0.077) [0.939–1.237]
	% reporting lack of food in last 7 days	1.000(0.008)[0.985–1.020]	0.995(0.008)[0.977–1.013]	0.995(0.009) [0.978–1.013]	0.995 (0.009) [0.978–1.013]	0.995(0.009) [0.978–1.013]

At the patient level, age, marital status, occupation, religion, the severity of complications following abortion, gestational age of pregnancy, gravidity, history of previous abortion, contraceptive use before index pregnancy, and fertility intentions at the time of pregnancy, were all significant factors associated with adoption of a highly effective contraceptive method.

Older women exhibited lower odds of adoption, while those who were previously married but were not married at the time of the interview had higher odds of adoption. Compared to farmer or unskilled employment, women who were engaged in skilled income-generating activities, students, and unemployed women had lower odds of adoption. Muslims and Catholics had lower odds than non-religious women or women from other Christian denominations. Women who presented with moderately severe complications had higher odds of adoption while women with severe complications and those who presented with second-trimester abortions had lower odds of adoption of a highly effective contraceptive method. When we controlled for spatial random effects, the above patient-level characteristics did not show significant influence on the outcome. However, the odds of adoption were higher for patients with previous abortions and past experience with modern contraceptives (81.8% and 63.7%). Patients who wanted to delay or prevent future pregnancies had about 51.9% and 60.5% higher odds respectively of receiving a highly effective method.

At the facility level, women receiving PAC services in hospitals designated at Levels IV-VI had about 55.0% lower odds of receiving a highly effective contraceptive method compared to those treated for PAC in health centers and clinics (Levels II-III). For a facility with a separate procedure room where PAC services were managed for evacuation, PAC patients were 2.9 times as likely to receive a highly effective method compared with patients treated in facilities without such a procedure room, while patients treated in public facilities were 2.6 times as likely to receive a highly effective method compared to their counterparts in private facilities. Being treated in facilities with obstetrics/gynecology specialists increased the odds of receiving a highly effective method by 67.2%, while patients treated in facilities that perceived PAC services as a burden to their healthcare provision had 36.2% higher odds of adoption. These two relationships were insignificant upon inclusion of spatial effects in the model.

For patients treated at facilities that offered maternity services in addition to PAC, the odds of adopting a highly effective method were lower by 57.7%. In all facilities, every additional modern contraceptive method available increased PAC patients’ odds of receiving a highly effective method by 19.1%.

**Fig 1:**
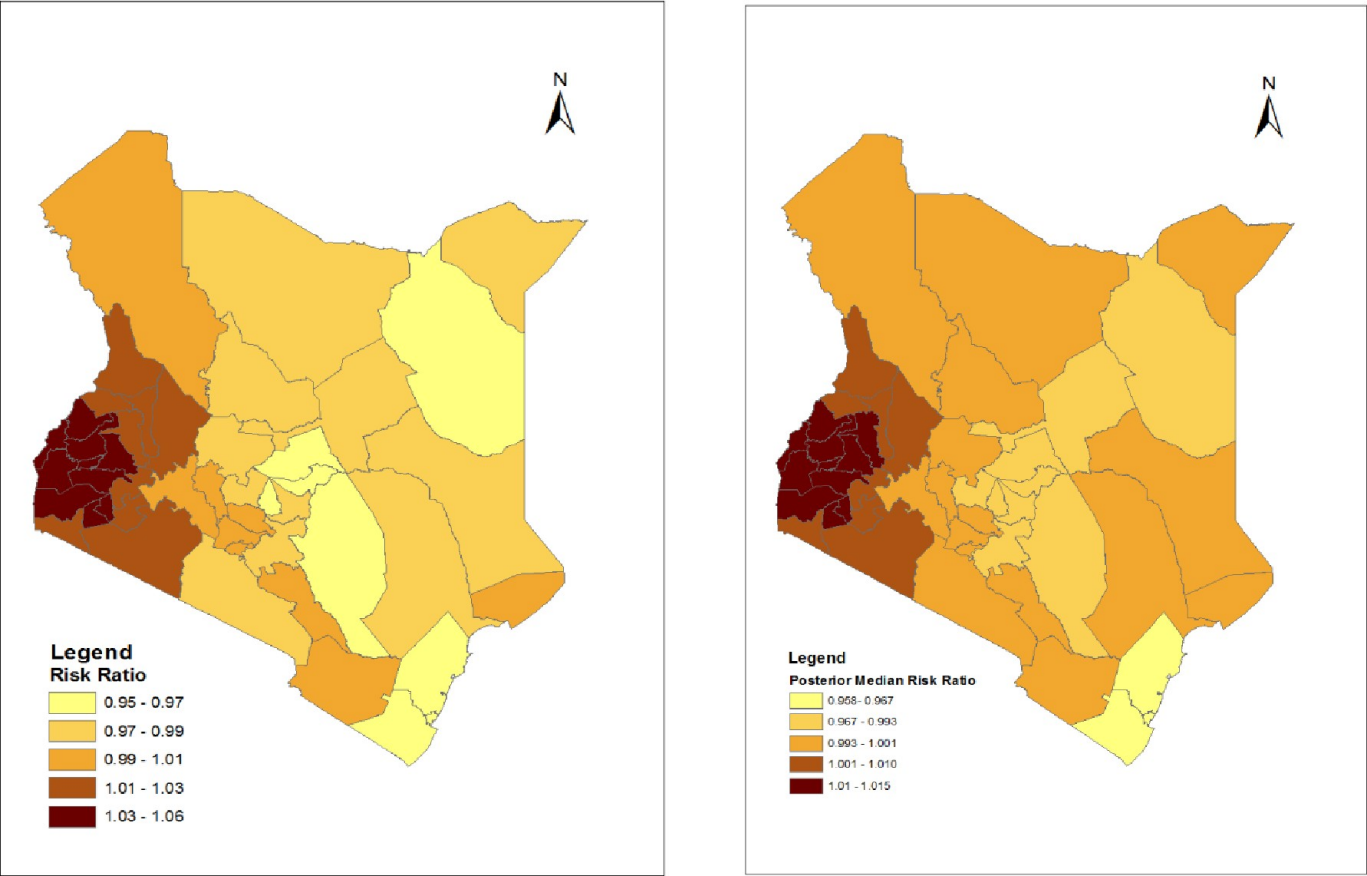
Structured and unstructured (convolution) spatial effect on odds of adoption of a highly effective contraceptive method- Posterior mean risk ratios.

**Fig 2:**
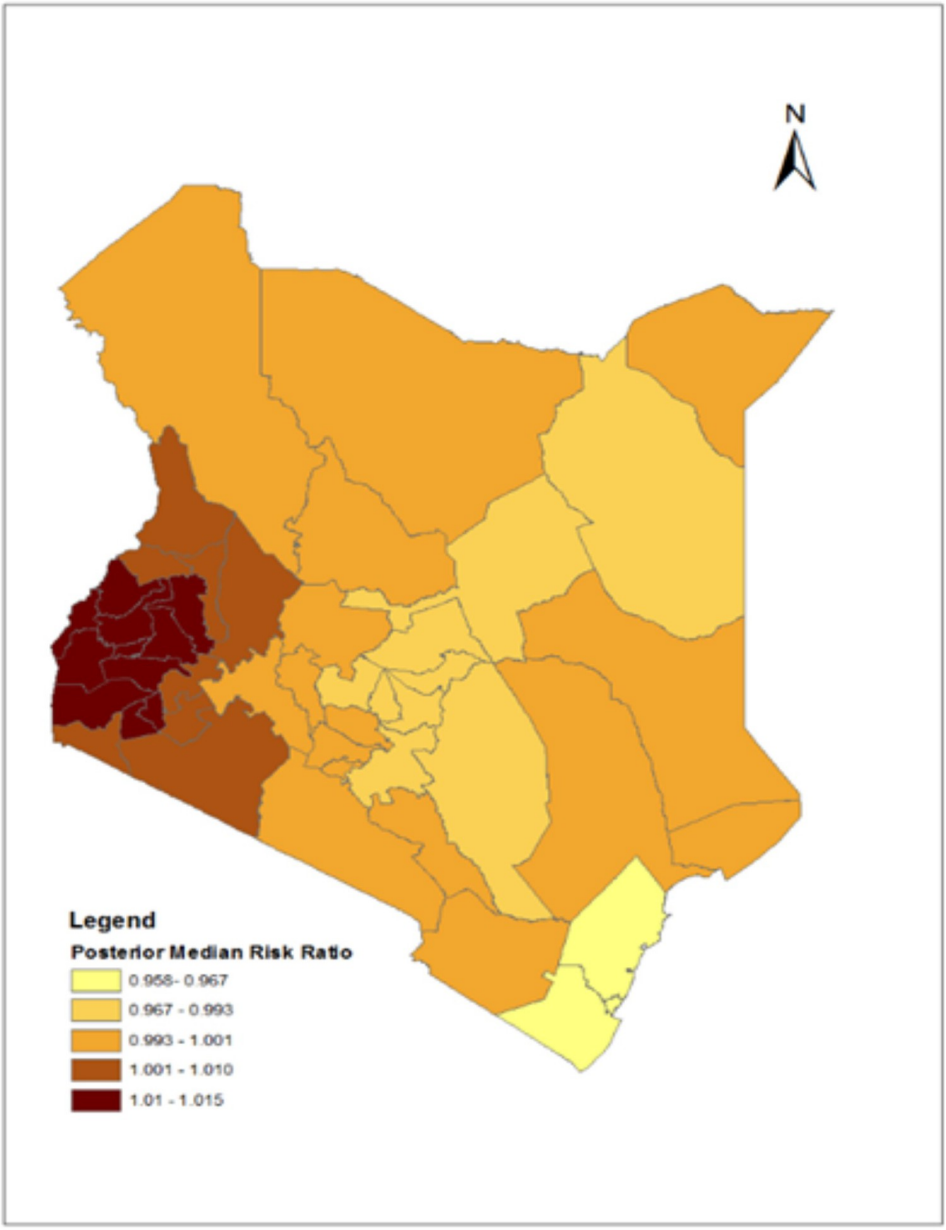
Structured and unstructured (convolution) spatial effect on odds of adoption of a highly effective contraceptive method- Posterior median risk ratios.

Figs 1 and 2 show the spatial distribution of the posterior means and medians respectively, of the odds of receiving a highly effective method overlaid on a map showing all counties in Kenya. The aim was to identify any geographical patterns in the county level effects on adoption of a highly effective contraceptive method.

The county posterior means and medians from the convolution model show that adoption of highly effective family planning for PAC patients was highest in the lake basin counties of Siaya, Homa Bay, Kisumu, Nyamira, Busia and Bungoma. Counties neighboring these also showed relatively high rates of adoption of highly effective contraception, and this holds for both the posterior means and the medians.

## Discussion

Post-abortion contraception plays a critical role in the provision of quality PAC services. This role is often compromised by not offering contraceptive methods that are highly effective in preventing unwanted and unintended pregnancies [5]. Although most studies have differed substantially, there is compelling evidence that effective post-abortion contraception can drastically reduce the risks of repeat abortions [17,31].

A significant number of patients observed during this study were treated in Level II facilities (37.6% in dispensaries). The majority (68.5%) of these patients were treated in government-owned facilities, signifying the role that the public healthcare system could play in providing quality PAC services in Kenya. As Thompson and colleagues [32] show, it is imperative that government policies on contraception have an impact on the level of quality for PAC services offered. Among these are policies that restrict access to certain services through certain regulatory requirements for training of service providers and the costs of service. At time of writing, only medical doctors, gynecology specialists, clinicians or registered comprehensive nurses are permitted to offer PAC service. This restricts access to quality PAC services in Level II facilities [33], even though a significant proportion of women receiving PAC were treated in these lower-level facilities. In our case, private facilities lagged behind public facilities in the provision of highly effective contraception.

The capacity of service providers to offer PAC including effective contraceptive counseling is essential in adhering to the principles of quality PAC. The average duration of service of the main PAC provider in the facility was reasonably high, with signs that could translate to more women adopting long-acting contraceptive methods post-care, even though this was not significant. We established that PAC trained staff were not always available to offer PAC services, indicating that effective contraception does not necessarily depend on the training of healthcare providers, but also ensuring their availability for actual PAC service delivery in these facilities. In addition, specialist service providers play a relatively important role in the adoption of highly effective contraception [16]. The adoption of long-acting reversible and permanent contraceptive methods is dependent on the service provider’s capacity to offer these methods during PAC [34].

Service integration is a way to increase the adoption of contraceptive services, by providing these services to PAC patients in a manner that minimizes loss to follow-up [4]. In this study, we established low uptake of highly effective family planning methods in facilities that offered maternity services, indicating the need to identify areas of operational synergy between PAC, family planning and maternity care [35]. Similarly, we established that patients treated in larger hospitals (Level IV-VI) were less likely to receive effective contraceptive methods after PAC. It has been demonstrated elsewhere that integrating PAC services with family planning reduces unsafe abortion by eliminating the risk of repeat abortion and increasing adoption of more effective contraceptives [36], and this has been a particular challenge in large hospitals, mainly due to poor patient follow-up especially when PAC and FP services are not offered under the same roof [37]. At a more contextual level, this call for service, including maternity care, is demonstrated further by the negative association between the community level of ANC service utilization and individual’s likelihood of adopting a highly effective contraceptive method.

We demonstrated that improving the contraceptive options available for PAC patients was positively associated with the likelihood of women adopting highly effective contraceptive methods. The level of integration must be one that ensures that PAC patients are provided with contraceptives of their informed choice, and under the same roof, avoiding missed opportunities that occur when PAC and FP clinics are separate. This explains why PAC patients in larger hospitals were less likely to adopt highly effective contraceptives, compared to those served in lower-level facilities where these services are more likely collocated [36]. This was also demonstrated through the higher adoption of highly effective contraception in facilities with evacuation procedure rooms, where all PAC cases are managed, by improving the quality of service provider and patient interaction prior to, during and after the procedure [38].

Despite high adoption of contraception soon after treatment for PAC, methods such as condoms and pills, compared to longer-acting methods, fail on effectiveness and expose women to future unwanted pregnancies [39,40]. Together with the wide range of services available for PAC patient [41], women with improved decision-making capacity or independence, such as previous exposure to contraception, increased their adoption of highly effective family planning methods. Certain patient-related characteristics also influence this uptake, pointing to the importance of effective post-abortion contraceptive counseling that supports patients in making informed decisions on appropriate contraceptive methods. Religious affiliation, past exposure to contraceptive methods, and patient’s fertility desires all play important roles in contraceptive decision-making and should be considered during post-abortion contraceptive counseling. The adoption of a highly effective family planning method begs for a concerted effort to improve the quality of interaction between the service provider and the patient, in a manner that ensures the final decision made by the patient is informed and reduces future exposure to unintended and unwanted pregnancies.

The maps present some interesting findings on spatial effects on highly effective contraception. Recent national estimates of contraceptive prevalence have shown significant increases in the use of long-acting reversible contraceptives and this is corroborated by our findings. According to the Kenya Demographic and Health Survey 2014, Western and Nyanza regions had the highest rates of use of highly effective family planning, especially implants (15.2% and 12.4% respectively reported that they were using implants), higher than all other regions. These counties do not have the highest rates of IUCD prevalence, pointing to the key role of implants in the prevention of unwanted pregnancy [13]. There has been a major investment in FP programs in Western and Nyanza regions of Kenya, leading to improved contraceptive uptake [42–44]. In addition, these regions have experienced substantial investments in HIV/AIDS prevention and treatment in the past decade [45]. HIV/AIDS and FP services are being integrated in the region [46], explaining in part the high uptake of highly effective contraception.

The main limitation of this study relates to the design of the parent study on which the data are based. This study was facility-based and in most of the cases, data are not representative of the general population. This negatively affects the ability to generalize its findings to the general population of women with post-abortion complications, given the low utilization of healthcare services in Kenya.

The availability of abortifacients which have become more readily available in the drugs market [47] may have reduced the number of patients seeking care for induced abortion. At analysis, we could not establish the actual placement of patients’ place of residence and the role of distance to care in the relationship between patient and facility characteristics on one side, and the adoption of highly effective contraceptive methods on the other. Finally, data used in this study were collected by trained service providers during and after treatment for complications and responses may have been subject to provider bias, and to Hawthorne effects on service providers' participation in the study, with service providers acting correctly because they knew that they were being observed.

### Conclusion

Improving the reproductive health of PAC patients requires considerable efforts at policy, service delivery, and individual levels. These efforts must aim to empower patients to make an informed choice of whether to use contraception and to choose the type of contraception which best suits their reproductive needs. At the policy level, there is a need to focus on service provider skills that are sensitive to patients’ needs and ability to make decisions, and on barriers that influence choice. Widespread service provider empowerment through capacity building for PAC, infrastructural improvement, and contraceptive commodity options, and their secured availability, are paramount in ensuring that they can interact better with patients and can deliver quality PAC services. Together with their availability to offer PAC, service providers can achieve these desirable contraceptive outcomes through effective counseling and contraception to reduce the risks of method failure. This can be attained by opting for methods whose effectiveness are less dependent on patient compliance, such as pills, condoms and injectable contraceptives, and focusing on methods with guaranteed effectiveness soon after a PAC procedure. At the patient level, effective counseling of PAC patients requires understanding the patient’s past experience with contraception and future fertility intentions and desires, in order to meet their needs more specifically. Finally, family planning integration with PAC will ultimately reduce missed opportunities, while promising improved reproductive health outcomes of post-abortion care patients. The potential relationship between the spatial effects on uptake of effective contraception, showing a higher uptake in regions with more health interventions in HIV/AIDS treatment and prevention presents an opportunity for further investigation.

## Appendix A: Model specification

### Appendix A1: A Model specification for ordered categorical data

Our outcome variable is defined as whether a PAC patient received a highly effective contraceptive method (2), or a less effective contraceptive method (1), or whether they did not receive any method at all (0), and is ordinal in nature. In addition, patients treated in a particular facility are more likely to have more similar characteristics compared to patients treated in other facilities. Similarly, facilities in one county are likely to have certain characteristics that make them more similar than facilities in other counties while counties close by are likely to exhibit similar characteristics compared to counties that are far apart. We use hierarchical ordered logit models in our analysis, with two levels of random variation by facility and county. The ordinal logit model is developed from the general form of the binomial models given as;

$$L\left(x_{ifj},y_{ifj}|p_{ifj}\right)=L\left\{Y_{ifj}=y_{ifj}\right\}=\left(\begin{array}{c} r_{ifj}\\ y_{ifj} \end{array}\right)p_{ifj}^{y_{ifj}}{\left(1-p_{ifj}\right)}^{r_{ifj}-y_{ifj}}$$ (1)

In ordinal logit regression, the event of interest is observing a particular contraceptive use or less.

Suppose k=0,1,2 are the ordered categories of contraceptive use, the odds for each category are defined as follows;

$$p_{1}=\text{prob(contraceptive adoption 0)/prob(contraceptive adoption>0)}$$

$$p_{2}=\text{prob(contraceptive adoption or 1)/prob(contraceptive adoption>1)}$$

$$p_{3}=\text{prob(contraceptive adoption or or 2)/prob(contraceptive adoption>2)}$$

In general, this can be described in the form

$$p_{k}=\text{prob(contraceptive adoption}\leq k\text{)/prob(contraceptive adoption>k)}$$ (2)

Let $y_{ij}$ denote the ordinal contraceptive use for patient *i* from a facility in county j. In general, the probability that a patient response will fall in a category higher than k is;

$$Pr\left(y_{ij}>k\;|\;X_{ij},k,\mu_{j}\right)=\;H\left(X_{ij}\beta +z_{ij}\mu_{j}-k_{k}\right)$$ (3)

Where j=1,2,3,…,47 are the counties consisting of $i\;=\;1,2,3,\ldots ,n_{j}$ patient observations in county j. The cut points k are labelled $k_{1},k_{2},k_{3},\ldots ,\;k_{k-1}$ for k possible outcomes. $X_{ij}$ are the fixed predictors in the model with regression coefficients β. These $X_{ij}$ predictors do not contain the constant term since the cut-off points absorb the effects of the constant term. The random effects $\mu_{j}$ are assumed to have a multivariate normal distribution.

Without loss of generality, the probability of an observation for an ordered logit model with county- level random effects $\mu_{j}$ can be defined as follows with the error term included;[48]

$$p_{ij}=Pr\left(y_{ij}>k\;|\;X_{ij},k,\mu_{j}\right)=Pr\left(k_{k-1}<n_{j}+\epsilon_{ij}\leq k_{k}\right)$$ (4)

Rearranging the terms in the above model gives;

$$p_{ij}=prob\left(k_{k-1}-n_{j}<\epsilon_{ij}\leq k_{k}-n_{j}\right)=prob\;\left(\epsilon_{ij}\leq k_{k}-n_{j}\right)-prob\;\left(\epsilon_{ij}\leq k_{k-1}-n_{j}\right)$$

Replacing the $Pr\;\left(y_{ij}>k\right)$ part in the above equation gives;

$$Pr\;\left(y_{ij}=k\;|\;k,\mu_{j}\right)=H\left(k_{k}-X_{ij}\beta -z_{ij}\mu_{j}\right)-H\left(k_{k-1}-X_{ij}\beta -z_{ij}\mu_{j}\right)$$ (5)

The above probability function can also be written in terms of latent linear response as;

yij*=Xijβ+zijμj+ϵijand

$$y_{ij}=\{\begin{array}{cc} 1 & if\;y^{*}{}_{ij}\leq k_{1}\\ 2 & if\;k_{1}\leq y^{*}{}_{ij}\leq k_{2}\\ 3 & if\;k_{2}\leq y^{*}{}_{ij}\leq k_{3}\\ & .\\ & .\\ & .\\ K & if\;k_{K-1}\leq y^{*}{}_{ij} \end{array}$$

Where the errors $\epsilon_{ij}$ follow a logistic distribution, gives the ordered logit model and are independent of the random effects. Thus in terms of the binomial distribution and logit model can be written as;

$$Pr\left(y_{ij}=k\;|\;k,\;\mu_{j}\right)=\frac{1}{1+exp\left(-k_{k}+n_{ij}\right)}-\frac{1}{1+exp\left(-k_{k-1}+n_{ij}\right)}$$ (6)

Where $n_{ij}\;=\;x_{ij}\beta +z_{ij}\mu_{j},\;k_{0}\;$is defined as -∞ and $k_{k}\;$is defined as +∞.

Within the jcounties, j=1,2,3,…,m=47 the outcome variable has a conditional distribution of $y_{ij}\;=\;\left(y_{j1},\;y_{j2},y_{j3},\;\ldots ,y_{jn_{j}}\right)'$ given the county random effects $\mu_{j}$, which is defined as;

$$L\left(y_{j}\;|k,u_{j}\right)=\prod_{i=1}^{n_{j}}p_{ij}^{I_{k}\left(y_{ij}\right)}$$ (7)

And taking the natural logarithms and replacing $p_{ij}$ with its likelihood is expressed as follows’

$$\text{Log}\;\left\{L\;\left(y_{j}\;|k,u_{j}\right)\right\}=\text{exp}\overset{n_{j}}{\underset{i=1}{\sum }}\left\{I_{k}\left(y_{ij}\right)\text{ln}\left(p_{ij}\right)\right\}$$

$$=exp\sum_{i=1}^{n_{j}}\left\{I_{k}\left(y_{ij}\right)\;ln\;\left[\frac{1}{1+exp\left(-k_{k}+n_{ij}\right)}-\frac{1}{1+exp\left(-k_{k-1}+n_{ij}\right)}\right]\right\}$$ (8)

Where;

Ik(yij)={1ifyij=k0otherwiseandnij=Xijβ+zijμj

The full log-likelihood function is defined as;

$$\text{Log}\;\left\{L\left(y_{ij}\;|k,u_{j}\right)\right\}=exp\sum_{i=1}^{n_{j}}\left\{I_{k}\left(y_{ij}\right)\;ln\;\left[\frac{1}{1+\text{exp}\left(-k_{k}+n_{ij}\right)}-\frac{1}{1+\text{exp}\left(-k_{k-1}+n_{ij}\right)}\right]\right\}$$ (9)

The prior distribution of $\mu_{j}$ is multivariate normal with mean zero and a 47 by 47 variance matrix $\sum_{47\times 47}$. Thus the likelihood contribution for each county is obtained by integrating out the random effects $\mu_{j}$ from the joint density $L\;\left(y_{j}|k,\mu_{j}\right)$ and can be given as;

$$L_{j}\left(\beta ,k,\sum \right)={\left(2\pi \right)}^{-q/2}\;{\left|\sum \right|}^{-1/2}\int L\;\left(y_{j}\;|k,\mu_{j}\right)\text{exp}\left(-\frac{u_{j}^{'}\sum^{-1}\mu_{j}}{2}\right)d\mu_{j}$$

$$={\left(2\pi \right)}^{-q/2}|\sum {|}^{-1/2}\int exp\{h(\beta ,k,\sum ,\mu_{j})\}d\mu_{j}$$ (10)

Where

$$h\left(\beta ,k,\sum ,\mu_{j}\right)=\overset{n_{j}}{\underset{i=1}{\sum }}\left\{I_{k}\left(y_{ij}\right)\text{ln}\left(p_{ij}\right)\right\}-\frac{\mu_{j}^{'}\sum^{-1}\mu_{j}}{2}$$

The above equation has no closed form. It can therefore be approximated for the maximum likelihood estimation using mean–variance adaptive Gauss–Hermite quadrature and Laplacian approximation crossed random-effects models.

The initial Bayesian models were fitted within Stata 14, but Stata does not cater for spatial random effects. Therefore, this is extended to the Bayesian framework such that the link function predictor $n_{ij}$ is extended such that;

nij*=Xijβ+zijμj+ϵij,lettingzijϑj=υj+ϕj

The spatial Bayesian model is defined as follows;

Posterior[p(parameters|data)]αLikelihood×Priors

The full conditional distribution for our model can be expressed as

$$p\left(\Phi \equiv \left\{\beta ,\phi ,v,X_{ij}\right\}|y_{ij}\right)\;\propto \;p(\Phi \;|y_{ij})=L\left(y_{ij},X_{ij}\;|\Phi \right)\times p\left(\beta_{k}\right)\times p\text{(}\phi_{j}\;|\tau_{c})\times p(\vartheta_{j}\;|\;\tau_{h})$$

$$=L\left(y_{ij}|\Phi \right)\times p\left(\beta_{k}\right)\times \;p\text{(}\phi_{j}\;|\tau_{c})\times p\left(\tau_{c}\right)\times p(\vartheta_{j}\;|\;\tau_{h})\times p\left(\tau_{h}\right)$$ (11)

The prior for the beta coefficients in the model is $\beta_{k}\begin{array}{c} iid\\ \sim \end{array}N(\mu_{\beta },\sigma_{\beta }^{2})$, thus

$$p\left(\beta_{k}\right)=\frac{1}{\sqrt{2\pi \sigma_{\beta }^{2}}}exp\left[-\frac{1}{2}{\left[\frac{\beta_{k}-\mu_{\beta }}{\sigma_{\beta }}\right]}^{2}\right]$$

The unstructured random effects $p\left(\vartheta_{j}|\tau_{h}\right)$, where $\vartheta_{j}\begin{array}{c} iid\\ \sim \end{array}N\left[0,\;\frac{1}{\tau_{h}}\right]$ with a Gamma function prior used $\tau_{h}\sim Gamma(\alpha_{h},\;\beta_{h})$ as shown by Banerjee [49]

With a Gamma function prior used $\tau_{h}\sim Gamma(\alpha_{h},\;\beta_{h})$, then

$$p(\tau_{h})=\frac{\beta_{h}{}^{\alpha_{h}}}{\Gamma \left(\alpha_{h}\right)}\tau_{h}^{\alpha_{h}-1}\exp\left(-\beta_{h}\tau_{h}\right)\;\text{for}\;\alpha_{h}>0;\beta >0$$

$$\text{Therefore}\;p\left(\vartheta_{j}|\tau_{h}\right)=\frac{1}{\sqrt{\frac{2\pi }{\tau_{h}}}}exp\left[-\frac{1}{2}\;{\left[\frac{\vartheta_{j}-0}{\frac{1}{\sqrt{\tau_{h}}}}\right]}^{2}\right]$$

$$p\left(\vartheta_{j}|\tau_{h}\right)\times p\left(\tau_{h}\right)\propto \;\tau_{h}^{\alpha_{h}-1}\text{exp}\left(-\beta_{h}\tau_{h}\right)\times exp\left[-\frac{1}{2}\;{\left[\frac{\vartheta_{j}-0}{\frac{1}{\sqrt{\tau_{h}}}}\right]}^{2}\right]$$ (12)

A Continuous Autoregressive (CAR) prior is used for the structured spatial random effects $\vartheta_{j}\sim CAR(\tau_{c})$. The CAR prior is given as $\phi_{j}|\phi_{i}\;\neq i,\tau_{c}\sim N\;\left[\frac{\phi_{j}}{{\bar{\phi }}_{j}},\;\frac{1}{\tau_{c}m_{j}}\right]$ and a conjugate hyper prior of $\tau_{c}\sim Gamma(\alpha_{c},\beta_{c})$ was assumed. Thus, as shown in Banerjee and Lawson [27,50];

$$p(\phi_{j}|\tau_{c})=\frac{1}{\sqrt{\frac{2\pi }{\tau_{c}m_{j}}}}exp\left[-\frac{1}{2}{\left[\frac{\phi_{j}-\frac{\phi_{j}}{{\bar{\phi }}_{j}}}{\sqrt{\tau_{c}m_{j}}}\right]}^{2}\right]$$ (13)

Within the spatial random effects model, it was considered that as much as facilities within one county are more similar to each other than to facilities in other counties, counties near each other are likely to have higher correlations than counties that are far away from each other. The likelihood of the neighbouring counties is therefore given as;

$P\left(\phi_{j}|\tau_{c}\right)\propto \exp\left\{-\frac{\tau_{c}}{2}\sum_{i=1}^{n_{j}}w_{ij}{\left(\phi_{j}-\phi_{i}\right)}^{2}\right\}$ where $w_{ij}\;$denotes the adjacency matrix and the subscript ij shows that the county jis a neighbour of county i and $m_{j}$ is the number of neighbours for county j. A Gamma distribution of the conjugate hyper prior $\tau_{c}\sim Gamma(\alpha_{c},\beta_{c})$ is used and whose distribution function can be shown as;

P(τc)=(βc)αcΓ(αc)τcαc−1exp(−βcτc),forα>0;β>0,thus;

sincep(ϕj|τc)∝exp{−τc2∑i=1njwij(ϕj−ϕi)2},then

$$p\left(\phi_{j}|\tau_{c}\right)\times p\left(\tau_{c}\right)\;\propto \;\exp\left\{-\frac{\tau_{c}}{2}\sum_{i=1}^{n_{j}}w_{ij}{\left(\phi_{j}-\phi_{i}\right)}^{2}\right\}$$ (14)

Combining the equations above, a full conditional likelihood function for the spatial structured and unstructured model can be given as;

$$P\left(\Phi |y_{ij}\right)=L\text{(}y_{ij},\;x_{ij}\;|\Phi )\times p\left(\beta_{k}\right)\times p(\phi_{j}|\tau_{c})\times p(\vartheta_{j}|\tau_{h})$$

$$P\left(\Phi |y_{ij}\right)=exp\sum_{i=1}^{n_{j}}I_{k}\left(y_{ij}\right)\text{ln}\left[\frac{1}{1+\exp\left(-k_{k}+n_{j}\right)}-\frac{1}{1+\exp\left(-k_{k-1}+n_{j}\right)}\right]\times \frac{1}{\sqrt{2\pi \sigma_{\beta }^{2}}}\exp\left[-\frac{1}{2}{\left[\frac{\beta_{k}-\mu_{\beta }}{\sigma_{\beta }}\right]}^{2}\right]\times \tau_{r}^{\alpha_{r-1}}\exp\left(-\beta_{r}\tau_{r}\right)\times \tau_{h}^{\alpha_{h-1}}\exp(-\beta_{h}\tau_{h})\times exp\left[\frac{1}{2}{\left[\frac{\vartheta_{j}-0}{\frac{1}{\sqrt{\tau_{h}}}}\right]}^{2}\right]\times \text{exp}\left\{-\frac{\tau_{c}}{2}\;\sum_{i=1}^{n}w_{ij}{\left(\phi_{j}-\phi_{i}\right)}^{2}\right\}\tau_{c}^{\alpha_{c-1}}\text{exp}\left(-\beta_{c}\tau_{c}\right)$$ (15)

The above equation has no closed form and has to be approximated. The approximation of the parameters was done using Markov Chain Monte-Carlo (MCMC) with Metropolis-Hastings algorithms approach. For the bivariate models in Stata, a *burnin* and thinning of 1000 and 10 respectively were used, while the multivariable model as well as the structured and spatial models were fitted in WINBUGS. In this algorithm, *burnin* removes unstable values while *thinning* reduces the autocorrelation.

### Goodness of fit

Goodness of fit for all ordered logit models presented in this paper was evaluated by comparing the Deviance Information Criteria (DIC) value for each model as defined in [24]

$DIC=D\left(\;\bar{u}\right)+\;p_{D}$ where $D\left(\;\bar{u}\right)$ is the posterior deviance expectation with $p_{D}$ effective parameters. The model that produced the smallest DIC value was considered to be better than its comparators with higher DIC.

## Appendix B: Codes for the different analysis methods used in this study

### Appendix B1:- STATA Bayesian MEOLOGIT models

#### B11. Bivariate patient level characteristics

foreach x in age4_2 age4_3 marital_2 marital_3 education_2 education_3 education_4 occupation_2 occupation_3 occupation_4 religion_2 religion_3 religion_4 severity_2 severity_3 gestation_2 gravidity_2 gravidity_3 prev_abortion_2 modern_2 wanted_2 wanted_3 referred_2 lev46_2 public_2 evacuation_2 obygyn_2 maternity_2 problem_2 strained_2 method_count qual_index unmetneedfp fpradio3months ancskillprov tt2 calloc2 lackf7days {

set seed 1234

bayes, mcmcsize(1000) burnin(1000) thinning(10): meologit contr2 `x' || county: || facility:, level(97.5) or

bayesgraph diagnostics _all, saving(diagnostics_`x')

}

#### Appendix B12. Multivariable model

bayes, mcmcsize(10000) burnin(1000) thinning(100): meologit contr2 age4_2 age4_3 marital_2 marital_3 education_2 education_3 education_4 occupation_2 occupation_3 occupation_4 religion_2 religion_3 religion_4 severity_2 severity_3 gestation_2 gravidity_2 gravidity_3 prev_abortion_2 modern_2 wanted_2 wanted_3 referred_2 lev46_2 public_2 evacuation_2 obygyn_2 maternity_2 problem_2 strained_2 method_count qual_index unmetneedfp fpradio3months ancskillprov tt2 calloc2 lackf7days || county: || facility:, level(97.5) or

bayesgraph diagnostics _all, saving("D:\PhD\PhD Paper II- Postabortion Contraception\Data and analysis\September 2017\Graphs\full_model_diagnostics")

### Appendix B2:- Unstructured random effects model

model {

for (i in 1:N) {# open the loop

       contra_n[i] ~ dcat (p[i,])

      p[i, 1] <- 1/ (1 + exp (-cA[1] + mu[i]))

      p[i, 2] <- 1/ (1 + exp (-cA[2] + mu[i])) - 1/ (1 + exp (-cA[1] + mu[i]))

      p[i, 3] <- 1–1/ (1 + exp (-cA[2] + mu[i]))

D[i] <- m[1]*equals(education[i],1) + m[2]*equals(education[i],2) + m[3]*equals(education[i],3) +m[4]*equals(occupation[i],2) + m[5]*equals(occupation[i],3) + m[6]*equals(occupation[i],4) +

m[7]*equals(severity[i],2) + m[8]*equals(severity[i],3) + m[9]*equals(gestation[i],2) + m[0]*(referred[i]) + m[11]*(public[i]) + m[2]*(obygyn[i])

E[i] <- q[1]*(problem[i]) + q[2]*(strained[i]) + q[3]*(lnpacstafftrained[i]) + q[4]*(qual_index[i]) + q[5]*(unmetneedfp[i]) + q[6]*(fpradio3months[i]) + q[7]*(ancskillprov[i]) + q[8]*(tt2[i]) + q[9]*(lackf7days[i])

A[i] <- s[1]*(lev46[i]) + s[2]*(evacuation[i]) + s[3]*(maternity[i]) +s[4]*(method_count[i]) + s[5]*(calloc2[i])

mu[i] <- b[1]*equals(age4[i],2) + b[2]*equals(age4[i],3) + b[3]*equals(marital[i],2) +b[4]*equals(marital[i],3) +b[5]*equals(religion[i],2) + b[6]*equals(religion[i],3) +b[7]*equals(religion[i],4) +b[8]*equals(gravidity[i],2) + b[9]*equals(gravidity[i],3) + b[10]*(prev_abortion[i]) +b[11]*equals(modern[i],1) + b[12]*equals(wanted[i],2) + b[13]*equals(wanted[i],3) + b[14]*equals(wanted[i],9) + A[i] + D[i] +E[i] +u[county[i]]+ f[facility[i]]

       }

for (k in 1:47) {

u[k] ~ dnorm(0, tau.h)

           }

for (i in 1:14) {

b[i]~dnorm(0,0.001)

              }

for (i in 1:5) {

s[i]~dnorm(0,1.0E-6)

              }

for (i in 1:9) {

q[i]~dnorm(0,1.0E-6)

              }

for (i in 1:12) {

m[i]~dnorm(0,1.0E-6)

              }

tau.b ~ dgamma(0.5, 0.0005)

sigma.b <- sqrt(1/tau.b)

tau.h ~ dgamma(0.5, 0.0005)

sigma.h <- sqrt(1/tau.h)

for (j in 1:281) {

   f[j] ~ dnorm(0.0,tauv)

   }

  cA[1] <- g[1]

   cA[2] <- g[1] + g[2]

   g[1] ~dnorm(0,0.01)

   #g[2] ~dnorm(0,0.01)

   g[2] ~ dgamma(0.001, 0.001)

   tauv ~ dgamma(0.001, 0.001) # prior structured

    }

### Appendix B3:- Structured random effects model

model {

for (i in 1:N) {# open the loop

       contra_n[i] ~ dcat (p[i,])

      p[i, 1] <- 1/ (1 + exp (-cA[1] + mu[i]))

      p[i, 2] <- 1/ (1 + exp (-cA[2] + mu[i])) - 1/ (1 + exp (-cA[1] + mu[i]))

      p[i, 3] <- 1–1/ (1 + exp (-cA[2] + mu[i]))

D[i] <- m[1]*equals(education[i],1) + m[2]*equals(education[i],2) + m[3]*equals(education[i],3) +m[4]*equals(occupation[i],2) + m[5]*equals(occupation[i],3) + m[6]*equals(occupation[i],4) +

m[7]*equals(severity[i],2) + m[8]*equals(severity[i],3) + m[9]*equals(gestation[i],2) + m[0]*(referred[i]) + m[11]*(public[i]) + m[12]*(obygyn[i])

E[i] <- q[1]*(problem[i]) + q[2]*(strained[i]) + q[3]*(lnpacstafftrained[i]) + q[4]*(qual_index[i]) + q[5]*(unmetneedfp[i]) + q[6]*(fpradio3months[i]) + q[7]*(ancskillprov[i]) + q[8]*(tt2[i]) + q[9]*(lackf7days[i])

A[i] <- s[1]*(lev46[i]) + s[2]*(evacuation[i]) + s[3]*(maternity[i]) +s[4]*(method_count[i]) + s[5]*(calloc2[i])

mu[i] <- b[1]*equals(age4[i],2) + b[2]*equals(age4[i],3) + b[3]*equals(marital[i],2) +b[4]*equals(marital[i],3) +b[5]*equals(religion[i],2) + b[6]*equals(religion[i],3) +b[7]*equals(religion[i],4) +b[8]*equals(gravidity[i],2) + b[9]*equals(gravidity[i],3) + b[10]*(prev_abortion[i]) +b[11]*equals(modern[i],1) + b[12]*equals(wanted[i],2) + b[13]*equals(wanted[i],3) + b[14]*equals(wanted[i],9) + A[i] + D[i] +E[i] +u[county[i]]+ f[facility[i]] + sp[county[i]]

    }

for (k in 1:47) {

u[k] ~ dnorm(0, tau.h)

            }

sp[1:47] ~ car.normal(adj[], weights[], num[], tau.b)

for (k in 1:sumNumNeigh)

            {

weights[k] <- 1

            }

for (i in 1:14) {

b[i]~dnorm(0,0.001)

            }

for (i in 1:5) {

s[i]~dnorm(0,1.0E-6)

            }

for (i in 1:9) {

q[i]~dnorm(0,1.0E-6)

            }

for (i in 1:12) {

m[i]~dnorm(0,1.0E-6)

            }

tau.b ~ dgamma(0.5, 0.0005)

sigma.b <- sqrt(1/tau.b)

tau.h ~ dgamma(0.5, 0.0005)

sigma.h <- sqrt(1/tau.h)

for (j in 1:281) {

    f[j] ~ dnorm(0.0,tauv)

    }

    cA[1] <- g[1]

    cA[2] <- g[1] + g[2]

    g[1] ~dnorm(0,0.01)

    g[2] ~ dgamma(0.001, 0.001)

    tauv ~ dgamma(0.001, 0.001)

      }

### Appendix B4:- Convolution (Structured and unstructured) random effects model

model {#open the model

for (i in 1:N) {

       contra_n[i] ~ dcat (p[i,])

      p[i, 1] <- 1/ (1 + exp (-cA[1] + mu[i]))

      p[i, 2] <- 1/ (1 + exp (-cA[2] + mu[i])) - 1/ (1 + exp (-cA[1] + mu[i]))

      p[i, 3] <- 1–1/ (1 + exp (-cA[2] + mu[i]))

D[i] <- m[1]*equals(education[i],1) + m[2]*equals(education[i],2) + m[3]*equals(education[i],3) +m[4]*equals(occupation[i],2) + m[5]*equals(occupation[i],3) + m[6]*equals(occupation[i],4) +

m[7]*equals(severity[i],2) + m[8]*equals(severity[i],3) + m[9]*equals(gestation[i],2) + m[10]*(referred[i]) + m[11]*(public[i]) + m[12]*(obygyn[i])

E[i] <- q[1]*(problem[i]) + q[2]*(strained[i]) + q[3]*(lnpacstafftrained[i]) + q[4]*(qual_index[i]) + q[5]*(unmetneedfp[i]) + q[6]*(fpradio3months[i]) + q[7]*(ancskillprov[i]) + q[8]*(tt2[i]) + q[9]*(lackf7days[i])

A[i] <- s[1]*(lev46[i]) + s[2]*(evacuation[i]) + s[3]*(maternity[i]) +s[4]*(method_count[i]) + s[5]*(calloc2[i])

mu[i] <- b[1]*equals(age4[i],2) + b[2]*equals(age4[i],3) + b[3]*equals(marital[i],2) +b[4]*equals(marital[i],3) +b[5]*equals(religion[i],2) + b[6]*equals(religion[i],3) +b[7]*equals(religion[i],4) +b[8]*equals(gravidity[i],2) + b[9]*equals(gravidity[i],3) + b[10]*(prev_abortion[i]) +b[11]*equals(modern[i],1) + b[12]*equals(wanted[i],2) + b[13]*equals(wanted[i],3) + b[14]*equals(wanted[i],9) + A[i] + D[i] +E[i] +u[county[i]]+ f[facility[i]] + sp[county[i]]

    }

for (k in 1:47) {

u[k] ~ dnorm(0, tau.h)

            }

sp[1:47] ~ car.normal(adj[], weights[], num[], tau.b)

for (k in 1:sumNumNeigh)

            {

weights[k] <- 1

            }

# All priors and hyperpriors:

#Priors for beta coefficients

for (i in 1:14) {

b[i]~dnorm(0,0.001)

            }

for (i in 1:5) {

s[i]~dnorm(0,1.0E-6)

          }

for (i in 1:9) {

q[i]~dnorm(0,1.0E-6)

            }

for (i in 1:12) {

m[i]~dnorm(0,1.0E-6)

            }

tau.b ~ dgamma(0.5, 0.0005)

sigma.b <- sqrt(1/tau.b)

tau.h ~ dgamma(0.5, 0.0005)

sigma.h <- sqrt(1/tau.h)

for (j in 1:281) {

    f[j] ~ dnorm(0.0,tauv)

    }

    cA[1] <- g[1]

    cA[2] <- g[1] + g[2]

    g[1] ~dnorm(0,0.01)

    #g[2] ~dnorm(0,0.01)

    g[2] ~ dgamma(0.001, 0.001)

    tauv ~ dgamma(0.001, 0.001) # prior structured

      }

## Supporting information

S1 File Supporting information. Authority to use data and questionnaires used to collect the data used in this study.
(ZIP)
