# Supplementary material for: Spatial and socio-economic correlates of effective contraception among women seeking post-abortion care in healthcare facilities in Kenya
Source: PLoS One. 2019 Mar 27;14(3):e0214049. doi: 10.1371/journal.pone.0214049 (PMC6436713; doi:10.1371/journal.pone.0214049)
Supplement: S1 File — Authority to use data and questionnaires used to collect the data used in this study. (ZIP) [file pone.0214049.s001.zip › Supporting information/S2 PONE-D-17-43970_Abortion Morbidity Survey Questionnaire.pdf]

Ministry of Medical Services (MOMS)

African Population and Health Research Centre (APHRC)

Division of Reproductive Health (DRH), Ministry of Public Health & Sanitation

STUDY OF ABORTION AND WOMEN'S HEALTH IN KENYA 2012

ABORTION CASE CAPTURE FORM

Kenya Medical Research Institute record number: 320

	**BACKGROUND INFORMATION**	**CODES**
K1	PROVINCE..	☐
K2	DISTRICT..	☐☐
K3	COUNTY...	☐☐
K4	HEALTH FACILITY NAME ________________	☐☐☐☐☐
K5	CLIENT IDENTIFICATION NUMBER..........................	☐☐☐☐☐
K6	DATE OF INTERVIEW..	☐ ☐ ☐ DD MM YY YY
K7	START TIME ☐☐☐☐ HH MM	END TIME ☐☐☐☐ HH MM
K8	NAME OF INTERVIEWER ________________	

<u>VERBAL CONSENT/ VERBAL ASSENT</u>

My name is -----------------------------------. During this month, we are conducting a study about post abortion care; and as we examine you and treat you, some of the information obtained will be used for research purposes. This information will be anonymous and no reference will be made to you whatsoever. The research work will also be confidential, and aggregate information collected will help the government to understand the magnitude of this problem in Kenya.

K9	RESULT OF INTERVIEW	1. COMPLETE..	☐
		2. REFUSED..	☐
		3. INCOMPLETE....................................	☐
		4. NOT AVAILABLE FOR INTERVIEW..........	☐
K10	COMMENTS:		

____

____

____

Section I: SOCIO-DEMOGRAPHICS OF THE CLIENT

This form should be completed for each case that presents to the facility seeking post abortion/abortion care during regular working hours as well as off working hours . All items in the form are very essential and should be filled accordingly. Make sure this form is available to all providers in your facility.

	QUESTIONS	RESPONSES	SKIPS
101	Age of client on her last birthday<br>**Record in completed years**<br><br>*If woman cannot remember her age at last birthday,*<br>**Ask her year of birth and calculate age.**	☐☐ Years Old<br><br>☐☐☐☐ Year of birth	
102	Regular Residence of client<br><br>**Tick only one response**	Urban……………………………… 1<br>Rural……………………………… 2<br>Other………………………….. 6	
103	Current marital status of client<br><br>**Tick only one response**	Never married/ never lived together…. 1<br>Married…………………………… 2<br>Living together…………………… 3<br>Separated/Divorced/widowed….. 4	
104	Highest level of education attained by client<br><br>**Tick only one response**	No education………………………. 1<br>Incomplete primary………………. 2<br>Completed primary………………. 3<br>Incomplete secondary…………… 4<br>Completed secondary…………... 5<br>College (mid-level)………………... 6<br>University…………………………. 7	
105	Religious affiliation of client<br><br>**Tick only one response**	Roman Catholic…………………… 1<br>Other Christian……………………. 2<br>Muslim……………………………… 3<br>No religion…………………………. 4<br>Other ………………..……………… 6<br>(specify) ____________	
106	Occupation of client<br><br>*Indicate what kind of work client mainly does*<br>**Tick only one response**	Farming…………………………… 01<br>Clerical……………………………. 02<br>Sales and services………………. 03<br>Skilled manual……………………. 04<br>Unskilled manual…………………. 05<br>Student……………………………. 06<br>House wife………………………… 07<br>Professional/technical/managerial 08<br>Unemployed………………………. 09<br>Other ……………………………… 96<br>(specify) ____________	

Section II: REPRODUCTIVE HISTORY OF THE CLIENT

	QUESTIONS	RESPONSES	SKIPS
201	**Total number of all pregnancies**<br><br>Please ***ask*** the client for all pregnancies she has had including the one she is seeking care for.	*(number)* ____________	
202	**Total number of live births**<br><br>Please ***ask*** the client for all live deliveries she has experienced.	*(number)* ____________	
203	**Total number of currently living children**<br><br>Please ***ask*** the client for all living children she has.	*(number)* ____________	
204	**Total number of previous spontaneous miscarriages.**<br><br>Please ***ask*** the client for how many miscarriages/spontaneous abortions she had before **excluding the one she is seeking care now**	*(number)* ____________	
205	**Total number of previous induced abortions**<br><br>Please ***ask*** the client for how many induced abortions she had before **excluding the one she is seeking care now.**	*(number)* ____________	
206	Was client using any form of contraception/ family planning method to prevent the current pregnancy that she is seeking care for?	YES………………………….. …………….. 1<br>NO………………………………………….. 2	→ 208
207	If yes, what type of contraceptives was the client using?<br><br>**Tick all that apply**	Pills…………………………………………. A<br>Injections……………………………….. B<br>Implants…………………………………… C<br>Female sterilization……………………..… D<br>Male sterilization………………………... E<br>IUD…………………………………………. F<br>Male condom…………………………..….. G<br>Female condom……………….……….. H<br>Diaphragm……………………...…….. I<br>Foam/jelly……………………………...….. J<br>Rhythm method……………………..…. K<br>Lactational Amenorrhea(LAM)……. L<br>Withdrawal method…………………... M<br>Emergency contraception………. N<br>Patch.. O<br>Other ……………….………. X<br>(specify) ____________	
208	At the time client became pregnant with this current pregnancy she is seeking care for did she want to become pregnant **then**, did she want to wait until **later**, or did she **not want** to have any more children at all?	Wanted **then**………………………….. 1<br>Wanted **later**…………………………….. 2<br>Did **not want at all**…………………... 3<br><br>Don't know………………………… 8	

Section III: CLINICAL HISTORY AND PHYSICAL EXAMINATION FINDINGS

A. History of present condition

	QUESTION	RESPONSES	SKIPS
301	Main reason for seeking care<br><br>**Tick only one response**	Postabortion care………………………… 1<br><br>Request for termination on medical or other grounds……………… 2	
302	Gestational age from last menstrual period (LMP) in weeks	Weeks……………………… 1 [ ][ ]<br>Unknown LMP……………… 2	
303	Did the woman provide a history of interference with the continuation of the pregnancy.	YES……………………………………… 1<br>NO……………………………………… 2	→ 305
304	Please know that we are not interested in the name of the person but the position/cadre. This could be a doctor, nurse, relative….<br><br>Who assisted the woman to induce?<br>**TICK ALL THAT APPLY**	Doctor…………………………………… A<br>Clinical Officer………………………….. B<br>Nurse…………………………………… C<br>Trained Midwife………………………… D<br>TBA/Traditional healer…………….. E<br>Pharmacist/ dispenser………………… F<br>Self -induced…………………………… G<br>Relative/ Friend……………………… H<br>Other …………………………………… X<br>(specify) ____________	
	Please check if Q301 **is "1"** then ask the following questions (305-312); if the answer is **"2" skip to Q310**		
305	How long did it take for the client to realize that she needed medical care?<br><br>Please ***ask*** and **indicate number** of either hours, days, weeks or months	Hours……………………… 1 [ ][ ]<br>Days……………………… 2 [ ][ ]<br>Weeks…………………… 3 [ ][ ]<br>Months…………………… 4 [ ][ ]	
306	After they realized they had a problem, how long did it take for the client to decide to seek health care?<br><br>Please ***ask*** and **indicate number** of either hours, days, weeks or months	Hours……………………… 1 [ ][ ]<br>Days……………………… 2 [ ][ ]<br>Weeks…………………… 3 [ ][ ]<br>Months…………………… 4 [ ][ ]	
307	After she decided to seek health care, how long did it take to reach the facility?<br><br>Please **ask** and **indicate number** of either hours, days, weeks or months	Hours……………………… 1 [ ][ ]<br>Days……………………… 2 [ ][ ]<br>Weeks…………………… 3 [ ][ ]<br>Months…………………… 4 [ ][ ]	

308	Where did the client first seek care when she had the complication?<br><br>**TICK ONLY ONE**	Clinic/Dispensary………………………. A<br>Health Center/Nursing home…………. B<br>Hospital…………………………………. C<br>Health profession's home……………… D<br>TBA's /Traditional healer's………… E<br>Home ……………………………… F<br>Pharmacy/drug store…………………… G<br>Other ……………………………………. X<br>(specify) ____________________	
309	Who was the first provider to treat the client when she had the complication?<br><br>**TICK ONLY ONE**	Doctor……………………………………. A<br>Nurse……………………………………… B<br>Trained midwife…………………………. C<br>Clinical officer…………………………… D<br>CHW………………………………………. E<br>TBA/Traditional Healer………………… F<br>Drug vendor/ Pharmacist……………… G<br>Self……………………………………… H<br>Relative/friend…………………………. I<br>Other……………………………………. X<br>(specify) ____________________	
310	Was the client referred from elsewhere to this facility?<br><br>Please ***ask*** if client was referred from another provider to the current provider	Yes without referral note…………… 1<br><br>Yes with a referral note….. ………… 2<br><br>No……………………………………… 3	
311	Did the client receive any treatment before being referred to this facility?	YES……………………………………… 1<br>NO………………………………………. 2	
312	How long did the client take to seek care at this health facility from the time of referral **(for those referred)** ?<br><br>Please ***ask*** and **indicate number** of either hours, days, weeks or months	Hours……………………… 1 [ ][ ]<br>Days……………………… 2 [ ][ ]<br>Weeks…………………… 3 [ ][ ]<br>Months…………………… 4 [ ][ ]	
**B. Vital Signs**			
313	***A. Body temperature***<br>*(rounded to the tenth decimal point)*	1 ____________________ ^{0}C	
314	***B. Pulse rate***	2 ____________________ per min	
315	***C. Blood pressure***	3 ____________________ mmHg	

C. PHYSICAL/BI-MANUAL/VAGINAL EXAMINATION FINDINGS.

Note to provider: To answer the following questions (Q316 to Q324) you have to perform thorough physical examination including bi-manual pelvic examination and vaginal examination whenever possible using a speculum and note your findings accordingly.

316	Abdominal tenderness	YES………………………………………… 1<br>NO…………………………………………… 2	
317	Adnexual tenderness	YES………………………………………… 1<br>NO…………………………………………… 2	
318	Any form of foreign body/material in genital tract (vagina, cervix, uterus)	YES………………………………………… 1<br>NO…………………………………………… 2	
319	Vaginal bleeding	YES………………………………………… 1<br>NO…………………………………………… 2	
320	Offensive or foul smelling vaginal discharge?	YES………………………………………… 1<br>NO…………………………………………… 2	
321	Which of the following mechanical injuries did you detect?<br><br>**Tick all that apply**	Cervical laceration……………………… 1<br>Cervical tears…………………………… 2<br>Tenaculum bites of the cervix………… 3<br>Tears on the vulva and/or vagina…….. 4<br>None of the above……………………… 5	
322	Did you elicit/ observe any signs suggestive of any of the following complications<br><br>[Early physical/ clinical signs suggestive of any of the following complications]<br><br>**Tick all that apply**	Pelvic abscess………………………….. A<br>Local/pelvic peritonitis………………… B<br>Generalized peritonitis…………..…. C<br>Uterine perforation…………………..…. D<br>Gangrenous uterus……………….…… E<br>Gangrenous bowel…………………….. F<br>Sepsis…………………………………….. G<br>Shock……………………………………… H<br>Tetanus………………………………….. I<br>Other ……………………………..……… X<br>(specify) ____________________<br>None of the above……………………… Y	
323	Which of the following signs of organ/system failure did you find on examination?<br><br>Late physical/ clinical signs suggestive of any of the following complications<br><br>**Tick all that apply**	Respiratory distress syndrome………. A<br>Renal failure…………………..………… B<br>Liver failure……………………………… C<br>Cardiac failure……………………..……… D<br>Coma…………………………..………… E<br>Disseminated Intravascular coagulation (DIC)………………………. F<br>Others……………………………..……… X<br>(specify) ____________________<br>No sign of organ/system failure……… Y	
324	Best clinical estimate of gestation by trimester	≤ 12 weeks…………………………….... 1<br><br>> 12 weeks…………………………….... 2	

Section IV: DIAGNOSIS ON ARRIVAL TO THE FACILIY FOR THE CURRENT CARE

Note to provider: Answer the following question (Q401-Q402) based on your evaluation of the clinical history, physical, bi-manual and vaginal examination findings.

	QUESTIONS	RESPONSES	SKIPS
401	The diagnosis is:<br><br>**Tick only one response**	Inevitable abortion………………………… 1<br>Incomplete abortion……………………… 2<br>Missed abortion…………………………… 3<br>Complete abortion………………………… 4<br>Others ……………………………………… 6<br>(specify) ____________	
402	Is the abortion also septic?	YES…………………………………………… 1<br>NO…………………………………………… 2	

Section V: TREATMENT

	QUESTIONS	RESPONSES	SKIPS
501	Was any uterine evacuation procedure performed to treat the client for her presenting problem?	YES…………………………………………… 1<br>NO…………………………………………… 2 →	505
502	Describe the procedure<br><br>**Tick only one response**	Dilation and curettage (D&C)……………… 1<br>Manual vacuum aspiration(MVA)………… 2<br>Electric vacuum aspiration(EVA)………… 3<br>Medical abortion<br>(e.g. cytotec/misoprostol)………………… 4<br>Digital Evacuation.................................. 5<br>Forceps Evacuation................................ 7<br>Others ……………………………………… 6<br>(specify) ____________	
503	The evacuation procedure was performed primarily by:<br><br>**Tick only one response**	Ob/Gyn……………………………………… 1<br>Medical Officer/ GP………………………… 2<br>Clinical officer……………………………… 3<br>Nurse………………………………………… 4<br>Trained Midwife…………………………… 5<br>Other ………………………………………… 6<br>(specify) ____________	
504	Where was the evacuation procedure performed?	Operating theatre…………………………… 1<br>Outpatient department……………………… 2<br>MVA procedure room……………………… 3<br>General ward………………………….. 4<br>Labour ward………………………….. 5<br>Other………………………………………… 6<br>(specify) ____________	
505	Was the client provided any pain medication during the evacuation procedure?	YES…………………………………………… 1<br>NO…………………………………………… 2 →	507

506	What did the client receive?<br><br>**Tick all that apply**	General anesthesia………………………..… 1<br>Para cervical LA/ Para cervical block…………………... 2<br>Sedation…………………….…………..…… 3<br>Analgesics……………………….…………… 4<br>Verbocaine……………………….………… 5<br>Others……………………………...………… 6<br>(specify) ____________________	
507	Was the client given antibiotics during her current visit at this facility?	YES…………………………...……………… 1<br>NO……………………………….…………… 2	→ 509
508	Type of antibiotics given:	Oral only……………………………..……… 1<br>Parenteral only……………………………... 2<br>Combined (oral + parentral)……………… 3	
509	Was the client given intravenous fluids during her current visit at this facility	YES…………………………...……………… 1<br>NO……………………………….…………… 2	
510	Was the client given blood or blood products during her current visit at this facility?	YES…………………………...……………… 1<br>NO……………………………….…………… 2	
511	Was the client given oxytocics (oxytocin or ergometrine or misoprostol) after the uterine evacuation procedure?	YES…………………………...……………… 1<br>NO……………………………….…………… 2	

**Section VI: SURGICAL PROCEDURES FOR COMPLICATIONS**			
	**QUESTIONS**	**RESPONSE**	**SKIPS**
601	Were any of the following surgical procedures performed on the client?<br><br>**Tick all that apply**	Hysterectomy…………………………………… A<br>Salpingectomy………………………..………. B<br>Abscess drainage……………………..……… C<br>Repair of cervical tear……………………… D<br>Repair of vaginal tears……………………… E<br>Repair of vulva/perineal tears……………… F<br>Repair of perforated uterus………………... G<br>Repair of gut perforation…………………… H<br>Laparatomy…………………………………. I<br>Other………………………………………… X<br>(specify) ____________________<br><br>None………………………………………… Y	
**Section VII: POSTABORTION CONTRACEPTION.**			
701	Was the client counseled on contraception upon discharge?	YES……………………………...……………… 1<br>NO…………………………………..…………… 2	
702	Was the client given modern contraception on discharge?	YES……………………………...……………… 1<br>NO…………………………………..…………… 2	→ 704
703	What did the client receive?<br><br>**Tick all that apply**	Pills…………………………………………… A<br>Injections……………………………………. B<br>Implants………………………………………. C<br>Female sterilization………………………… D<br>IUCD…………………………………………. E<br>Male condom………………………………… F<br>Female condom……………………………... G<br>Diaphragm…………………………………… H<br>Foam/jelly……………………………………. I<br>Patch... J<br>Other ……………………………….……… X<br>(specify) ____________________	
704	Was the client referred elsewhere for modern contraception?	YES……………………………...……………… 1<br>NO…………………………………..…………… 2	
**Section VIII: OUTCOME OF CLINICAL MANAGEMENT OF CURRENT CARE**			
801	The outcome of current care was<br><br>*Comment could include the cause of leaving against medical advise or the condition of the woman when she left*	Discharged well……………………………… 1<br>Died………………………………………… 2<br>Still hospitalized....................................... 3<br>Absconded………………………………. 4<br>Left against medical advice………………. 5<br>comment ____________________<br><br>Referred to other facility…………………… 5<br>specify ____________________	
802	How long did the client stay in the facility for care?	Less than 12 hours……………………..… 1<br>12 to 24 hrs………………………………... 2<br>More than 24 hours………………………… 3	
