# Supplementary material for: Spatial and socio-economic correlates of effective contraception among women seeking post-abortion care in healthcare facilities in Kenya
Source: PLoS One. 2019 Mar 27;14(3):e0214049. doi: 10.1371/journal.pone.0214049 (PMC6436713; doi:10.1371/journal.pone.0214049)
Supplement: S1 File — Authority to use data and questionnaires used to collect the data used in this study. (ZIP) [file pone.0214049.s001.zip › Supporting information/S2 PONE-D-17-43970_Content-Permission-Form_ABS Data APHRC.pdf]

Request for Permission to Publish Content under CC-BY License

Dear Rights Holder or Representative,

I have submitted a paper for publication in a PLOS journal, and wish to include the content listed below in the paper. I'm hereby requesting your (or your company's or institution's) permission to include the content in my paper. Please note that all PLOS journals are published under a Creative Commons Attribution License (CC BY), which allows for unrestricted use and distribution, even commercial, as long as attribution is given to the creator or rights holder of the content. See https://creativecommons.org/licenses/by/4.0/.

To grant me permission to use the content in my PLOS paper, please fill in the information below and then scan the completed form and send it to me at my email address.

Thank you.

My name:

Michael M. Mutua

My email address:

mmutua@aphrc.org

Description of the content which I'm seeking permission to use (citation and/or title, and pasted screen shot, if applicable):

KENYA - The Epidemiology of Unsafe Abortions in Kenya, Abortion Incidence and Complication from Unsafe abortions in Kenya Data (Health Facility Survey and Prespective Morbidity Survey)

Link to the Content:

http://aphrc.org/catalog/microdata/index.php/catalog/39

* * *

On behalf of myself or the rights holder, I hereby grant the permission sought herein.

Signature of Party Granting Permission:

Date:

Printed Name and Title:

