# Supplementary material for: Spatial and socio-economic correlates of effective contraception among women seeking post-abortion care in healthcare facilities in Kenya
Source: PLoS One. 2019 Mar 27;14(3):e0214049. doi: 10.1371/journal.pone.0214049 (PMC6436713; doi:10.1371/journal.pone.0214049)
Supplement: S1 File — Authority to use data and questionnaires used to collect the data used in this study. (ZIP) [file pone.0214049.s001.zip › Supporting information/S2 PONE-D-17-43970_Health Facility Survey Questionnaire.pdf]

Ministry of Medical Services (MOMS) | African Population and Health Research Center (APHRC) | Division of Reproductive Health (DRH), Ministry of Public Health & Sanitation

STUDY OF POST-ABORTION AND WOMEN'S HEALTH IN KENYA

SURVEY OF HEALTH FACILITIES IN KENYA

Kenya Medical Research Institute record number: 320

CONSENT

My name is_____________. I am part of a team of researchers conducting a national study on post-abortion care in Kenya. The team comprise of researchers from the Ministry of Health (MoH) and African Population and Health Research Center (APHRC) among other partners. We would like to ask for your cooperation in collecting information on **post-abortion care** in Kenya. This research will provide information about reproductive health care and contribute to the improvement of maternal health in Kenya and beyond.

Your health facility has been randomly selected to be part of this study. Your responses about care provided in your facility will be completely confidential and will be used for research purposes only. No personal reference will be made to your participation in this study. Your responses and those of other health providers in Kenya will be used to describe the general picture of abortion care in Kenya. This interview will take 30-45 minutes of your time.

While we would like to note that your expertise and experience make your participation critical and your views valuable to us, we would also like to emphasize that your participation is absolutely voluntary. You are therefore at liberty to terminate the interview at any time if you do not want to continue. Please also note that non-participation or withdrawal from the interview will not prejudice you in any way.

Do you agree to proceed with the interview?

AGREE……………………………………. ☐

DO NOT AGREE……………………… ☐

Reason for refusal:____

Thank you

If you have any questions or complaints about the study; please call or write to one of the following people

1 Dr. Chimaraoke Izugbara, APHRC: Principal Investigator; Tel: 020-4001050; Email: cizugbara@aphrc.org

2 Dr. Bashir Mohammed, MoH: Co- Investigator, Tel: 0722318084; Email: dbashirim@yahoo.com

3 Dr. Simon Mueke, MoH: Co- Investigator, Tel: 020-717077; Email: simonmueke@gmail.com

Health Facility Information

K0

	**Attempts**				
	**1st**	**2nd**	**3rd**	**4th**	**5th**
	DD MM	DD MM	DD MM	DD MM	DD MM
**Date**					
**Outcome**					

Outcome Reasons

1= Achieved
2= Facility closed
3= Respondent not there
4= Respondent too busy to be interviewed
5= Respondent resheduled
6= Unable to reach facility
7=Security concerns
8= Other ________________________

K1 INTERVIEWER'S NAME: ______________________ **K2** INTERVIEWER'S CODE: [ ][ ][ ][ ]

BACKGROUND

K3 TIME STARTED: [ ][ ] hr [ ][ ] min

K4 DATE OF INTERVIEW: D D M M Y Y Y Y [ ][ ][ ][ ][ ][ ][ ][ ]

K5 HEALTH FACILITY NAME: ______________________

K6 HEALTH FACILITY CODE: [ ][ ][ ][ ][ ]

K7 PHYSICAL ADDRESS: ______________________

K8 PROVINCE: ______________________ [ ]

K9 COUNTY: ______________________ [ ][ ]

K10 DISTRICT: ______________________ [ ][ ][ ]

K11 LEVEL OF HEALTH FACILITY AS PER THE MASTERLIST

LEVEL................	**2**	LEVEL..................	**5**
LEVEL................	**3**	LEVEL..................	**6**
LEVEL................	**4**	MSI/FHOK............	7

K12 LEVEL OF HEALTH FACILITY AS PER THE RESPONDENT

LEVEL................	**2**	LEVEL..................	**5**
LEVEL................	**3**	LEVEL..................	**6**
LEVEL................	**4**	MSI/FHOK............	7

K13 TYPE OF HEALTH FACILITY

NATIONAL HOSPITAL....................	**01**	MATERNITY HOSPITAL..........................	**06**
PROVINCIAL HOSPITAL..................	**02**	HEALTH CENTRE..................................	**07**
DISTRICT HOSPITAL.....................	**03**	CLINIC..	**08**
SUB-DISTRICT HOSPITAL..............	**04**	DISPENSARY..	**09**
NURSING HOME...........................	**05**	OTHER (Specify) ____________________	**96**

**K14**	OWNERSHIP	Public	**01**
		Private for-profit	**02**
		Faith-based organization/mission	**03**
		Non-governmental organization (NGO)	**04**
		Community Based organization (CBO)	**05**
		Other (Specify)________________	**96**
**K15**	INTERVIEW OUTCOME:	Completed	**01**
		Refused	**02**
		Incomplete	**03**
		Heatlh facility not functional	**04**
		Other specify ________________	**06**

Module I: Background Information		
	**Questions and Instructions**	**Responses, Codes and Filters**
101	What is your profession at this facility?<br><br>**[Circle the category thst applies to respondent]**<br><br>DO NOT PROMPT	OB-GYN........ **01**<br>Medical Officer/GP........ **02**<br>Clinical Officer........ **03**<br>Trained Midwife........ **04**<br>Nurse........ **05**<br>Nurse Aide........ **06**<br>Other (Specify) ________ **96**
102	Respondent's sex	Male........ **1**<br>Female........ **2**
103	How old are you?	☐☐ years
104	What is your highest professional/academic qualification you have completed?	College (middle level)........ **1**<br>Degree........ **2**<br>Post graduate........ **3**<br>Other (Specify) ________ **6**
105	Whats your main responsibility at this facility?	Facility administrator........ **1**<br>Facility in-charge........ **2**<br>Unit in-charge........ **3**<br>Health care provider (non-administrative)........ **4**<br>Other (Specify)________ **6**
106	How many years have you worked in your current primary position?<br>**[If less than 1 year, fill 00]**	☐☐ Years
107	Which units does this facility have?<br><br>**[Read out all categories. Multiple responses are allowed]**	Outpatient (ambulatory)........ A<br>Inpatient unit........ B<br>Operating room/theater........ C<br>Evacuation room........ D<br>Gynaecological Ward........ E<br>Labor Ward........ F<br>Maternity Ward........ G<br>MCH unit........ H<br>FP unit........ I<br>Intensive care unit (ICU)........ J<br>Emergency ward........ K<br>Laboratory........ L<br>Pharmacy........ M<br>Other (specify) ________ X

108	Which of the following services does this facility provide?<br><br>**[Read out all categories. Multiple responses]**<br><br>**If facility does not provide PAC, END interview**	Specialized (OB-GYN)…………………………A<br>Maternity and delivery…………………………B<br>Post abortion care/ counseling………………C<br>Post-abortion contraceptive counselling……E<br>None of the above……………………………Y
109	How many people in this facility are trained to provide PAC services?	[ ][ ] Staff **[If 00 skip to Module 2]**
110	How often is a staff member trained in PAC available to attend to PAC patients?	Rarely……………………… **1**<br>Sometimes………………… **2**<br>Almost Always…………… **3**<br>Always……………………… **4**

Module II: Postabortion Care

Now, I would like to ask you some questions regarding **medical care for abortion patients treated at this facility, irrespective of whether the abortion was spontaneous or induced.** I will also ask about **the number of patients that are treated for such abortion complications at this facility.** By abortion complications, we are referring to those complications that are severe enough to **need treatment** in a health facility. Abortion complications, as defined here, include not only the **extremely serious cases** such as those with sepsis or a perforated uterus, but also those cases which are termed "incomplete abortions," which are usually identified by heavy bleeding, and which present a somewhat **less severe** health risk to the woman, but which, nevertheless, need treatment at a health facility. In answering the following set of questions concerning abortion complications, please keep this definition in mind.

[Note that the abortion complication questions relate to both spontaneous and induced abortions. You should reiterate this as often as possible while completing this section.]

201	On average, about how many deliveries take place at your facility each month?<br><br>**[If respondent can't answer for the total number in the year 2011]**	1 Deliveries per month ☐☐☐☐☐<br>**OR**<br>2 Total number of deliveries during the **2011** calendar year ☐☐☐☐☐
202	In which sections of this health facility are post-abortion patients treated?<br><br>**[Read out the list of wards and circle all that apply]**	Outpatient ward/Recovery room………………… **A**<br>Operating Room……………………………….. **B**<br>Evacuation Room……………………………… **C**<br>Gyneacological ward…………………………… **D**<br>Labor Ward……………………………………… **E**<br>Maternity ward…………………………………… **F**<br>General female ward…………………………… **G**<br>Intensive care unit (ICU)………………………… **H**<br>Emergency ward………………………………… **I**<br>Operating Room……………………………….. **J**<br>Other (specify) ____________________ **X**
203	In this facility, are **post-abortion care patients** treated as **outpatients** (they don't spend the night in the facility), **inpatients** (they spend at least one night in the facility) ?<br><br>**[Probe to make sure you are capturing all that apply]**	Outpatient…………………… A<br>Inpatient……………………… B
204	During **a typical** month, about how many such **post-abortion** care **patients** would you estimate are treated as outpatients at this facility as a whole? Please remember to include all **post-abortion care patients** whether they are due to spontaneous or induced abortions.<br><br>**[probe to elicit a response for a typical month; if respondent is not able to provide you with that estimate, then probe for the number of outpatients in a typical year. Specify that this is a full calendar year (i.e. from January to December). Please reiterate to the respondent that the number is both spontaneous and induced abortion patients and should take into consideration all wards of the facility. If a range is given, probe further for an average number]**	1 Number of outpatients in a **typical** month ☐☐☐☐☐<br>**OR**<br>2 Number of outpatients in a **typical** year ☐☐☐☐☐

205	In the **past** month, about how many **post-abortion care** patients were treated as **outpatients** in this facility as a whole (please remember to include all **post-abortion care patients**, whether they are due to spontaneous or induced abortions)<br><br>**[Probe to elicit a response for the past month; if respondent is not able to provide you with that estimate then probe for the number of outpatients during the year 2011. Specify that this is a full calendar year (i.e. from Jan to Dec). Please reiterate to the respondent that the number should take into consideration all wards of the facility. If a range is given, probe further for an average number]**	1 Number of **outpatients** in the **past** month<br>☐☐☐☐☐<br>**OR**<br>2 Number of **outpatients** in the **past** year **2011**<br>☐☐☐☐☐
[IF **Q203** INDICATES THAT INPATIENT **SERVICES ARE PROVIDED**, THEN ANSWER **Q206-207** IF NOT, **GO TO Q208**]		
206	During **a typical** month, about how many such **post-abortion care patients** would you estimate are treated as **inpatients** at this facility as a whole? Please remember to include all **post-abortion care patients** whether they are due to spontaneous or induced abortions<br><br>**[Please probe to elicit a response for a typical month; if respondent is not able to provide you with that estimate then probe for the number of inpatient on typical year. Specify that this is a full calendar year (from Jan to Dec). Please reiterate to the respondent that the number is for spontaneous and induced abortion patients, and should in to consideration all wards of the facility. If a range is given, probe further for an average number]**	1 Number of **inpatients** in an a **typical** month<br>☐☐☐☐☐<br>**OR**<br>2 Number of **inpatients** in a **typical** year.<br>☐☐☐☐☐
207	In the **past** month, about how many **post-abortion care patients** were treated as **inpatients** in this facility as a whole? Please remember to include all **post-abortion patients**, whether they are due to spontaneous or induced abortions<br><br>**[Interviewer: Please probe to elicit a response for the past month; if respondent is not able to provide you with that estimate, then probe for the number of inpatients during the year 2011. Please reiterate to the respondents that the number should take consideration all wards of the facility. If a range is given, probe further for an average number]**	1 Number of **inpatients** in the **past** month<br>☐☐☐☐☐<br>**OR**<br>2 Number of **inpatients** in the **past** year (**2011**)<br>☐☐☐☐☐
208	During **a typical** month, about how many **post-abortion care patients** would you estimate are **referred** from this facility as a whole? Please remember to include all **post-abortion care patients**, whether they are due to spontaneous or induced abortion.<br><br>**[Probe to elicit a response for a typical month; if respondent is not able to provide you with that estimate, then probe for the number of referrals in a typical year. Specify that this is a full calendar year (Jan-Dec). Please reiterate to the respondent that the number is for spontaneous and induced abortion patients, and should take into consideration all wards of the facility. If a range is given, probe further for an average number]**	1 Number of **referrals** in an **typical** month<br>☐☐☐☐☐<br>**OR**<br>2 Number of **referrals** in a **typical** year (**2011**)<br>☐☐☐☐☐

209	In the **past** month, about how many **post-abortion care patients were referred** from this facility as a whole? Please remember to include all **post-abortion care patients**, whether they are due to complications of spontaneous or induced abortions.<br><br>**[Please probe to elicit a response for the past month; if respondent is not able to provide you with that estimate, then probe for the number of referral during the year 2011. Please reiterate to the respondent that the number should take into consideration all wards of the facility. If a range is given, probe further for an average number]**	1 Number of **referrals** in the **past** month ☐☐☐☐☐<br><br>**OR**<br><br>2 Number of **referrals** in the **past** year **(2011)** ☐☐☐☐☐<br><br>**[IF Q208 AND Q209 BOTH EQUAL 0, SKIP TO 211]**
210	When a PAC case comes in and you determine it's too severe for you to handle, what services do you give before you refer them on?<br>**[Circle all that apply. Do not prompt]**	Stop the bleeding……………………… **A**<br>Provide antibiotics…………………… **B**<br>Provide pain killers…………………… **C**<br>Provide IV fluids for dehydration…… **D**<br>Provide counselling…………………… **E**<br>We do not refer patients………….. **F**<br>None of the above…………………… **Y**<br>Other (Specify) ________________ **X**
211	How many post-abortion care patient referrals do you **RECEIVE** from other facilities in a typical month?	1 Number of **referrals received in typical** month ☐☐☐☐☐<br><br>**OR**<br><br>2 Number of typical **referrals received** in the year 2011 ☐☐☐☐☐
212	**[Refer to the previous figures and fill in below before asking the respondent about the patient totals]**<br><br>So to confirm what you have just told me, in a **typical month** (or year), your facility treated<br><br>☐☐☐☐☐ Outpatients<br>☐☐☐☐☐ Inpatients<br><br>for abortion complications?<br><br>**[Please read out the total number of spontaneous and induced abortion patients seen at this facility in a typical month (Q204 and Q206)**<br><br>Is this number correct?<br>**[If correct, please insert again at right; if not then correct Q 204 and Q 206 and insert at right.]**	Summary per **month**<br><br>Outpatients…………… **1** ☐☐☐☐☐<br>Inpatients:…….……… **2** ☐☐☐☐☐<br>Total …….…………… **3** ☐☐☐☐☐<br><br>OR<br><br>Summary per **year**<br><br>Outpatients…………… **1** ☐☐☐☐☐<br>Inpatients:…….……… **2** ☐☐☐☐☐<br>Total …….…………… **3** ☐☐☐☐☐

213	**[Refer to the previous figures and fill in below before asking the respondent about the patient totals].**<br><br>So to confirm what you have just told me, in the **past month** (or **during the year** 2011) your facility treated:<br><br>☐☐☐☐☐ Outpatients<br>☐☐☐☐☐ Inpatients<br><br>for post-abortions complications?<br><br>**[Read out the total number of spontaneous and induced abortion patients seen at this facility in the past month (Q 205 and Q 207)].**<br><br>Is this number correct?<br>**[If correct, please insert again at right; if not then correct Q 205 and Q 207 and insert at right.]**	Summary in the **past month**<br>Outpatients…………… **1** ☐☐☐☐☐<br>Inpatients:……..……… **2** ☐☐☐☐☐<br>Total ………………….. **3** ☐☐☐☐☐<br><br>**OR**<br><br>Summary in the **past year (2011)**<br><br>Outpatients…………… **1** ☐☐☐☐☐<br>Inpatients:……..……… **2** ☐☐☐☐☐<br>Total ………………….. **3** ☐☐☐☐☐
214	What are the various procedures and measures used to treat abortion-complication patients at this facility?<br><br>**[Circle all applicable responses. Do not prompt respondent but probe to make sure you've captured all procedure types.]**<br>**Ask is there any other procedure?**	D & E (dilation and evacuation)……………….. **A**<br>D & C (dilation and curettage)…………………. **B**<br>MVA (manual vacuum aspiration)……………. **C**<br>EVA (electric vacuum aspiration)……………. **D**<br>Medical abortion (e.g. Cytotec/misoprostol)…. **E**<br>None…………………………………………. **F**<br>Other (*specify)* ______________________ **X**
215	Of these procedures, which is the most commonly used for treating patients with abortion complication at this facility?<br><br>**[Mark only one answer]**	D & E (dilation and evacuation)……………….. **01**<br>D & C (dilation and curettage)…………………. **02**<br>MVA (manual vacuum aspiration)……………. **03**<br>EVA (electric vacuum aspiration)……………. **04**<br>Medical abortion (e.g. Cytotec/misoprostol)…. **05**<br>Other (specify) ______________________ **96**<br>Not applicable…………………………………. **99**

Module III: Postabortion Counseling

301 To whom does this facility offer post-abortion contraceptive counseling?

[Circle all that apply. PROMPT]

1= Never/Rarely
2= Sometimes
3= Always
9= Not Applicable

		N/R	S	A	N/A
A	Everyone………………………….	1	2	3	9
B	Those who have many children..	1	2	3	9
C	Those who are married…………	1	2	3	9
D	Those who are of an older age…	1	2	3	9
X	Other (specify) ______________	1	2	3	9
	______________________________				

[IF ALWAYS TO EVERYONE, SKIP TO Q303]

302 For those patients to whom you don't offer post-abortion contraceptive counseling (i.e. rarely or sometimes), what are the reasons?

____
____
____
____

303 Do you think women treated for abortion complications **should** be given contraceptive counseling while still in the health facility?

Yes…………………… **1** → **[Go to 305]**
No…………………… **2**
Sometimes…………… **3** → **[Go to 305]**
Don't know/No opinion. **4** → **[Go to 305]**

304 Why do you think women treated for abortion complications should NOT receive family planning/contraceptive counseling?

____
____
____
____

305 What topics **do you generally** cover in contraceptive counseling for post-abortion care patients?

[Multiple responses allowed. Do not prompt]

Instructions on correct use of all methods………………… **A**
Instructions on available methods……………………….. **B**
Instructions on traditional methods only…………………… **C**
Advantages & disadvantages of each……………………… **D**
What to do in cases of method failure or forgetting pills… **E**
Abstinenance……………………………………………….. **F**
Other information you think should be included…………. **X**
(specify) ______________________________

306 What topics should be covered in the counseling on contraception for post-abortion patients?

[Multiple responses allowed. Do not prompt]

Instructions on correct use of all methods………………… **A**
Instructions on available methods……………………….. **B**
Instructions on traditional methods only…………………… **C**
Advantages & disadvantages of each……………………… **D**
What to do in cases of method failure or forgetting pills… **E**
Abstinence……………………………………………….. **F**
Other information you think should be included…………. **X**
(specify) ______________________________

307 How do you involve men in contraceptive selection?

____
____
____

308	Do you offer contraceptive methods on the premises of this facility?	Yes…………………**1**<br>No…………………**2** **[Go to Module 4]**
309	Which methods does your facility **commonly** offer to post-abortion care patients?<br><br>**[Do not prompt. Multiple responses allowed]**	Pills…………… **A**<br>Injectables…………… **B**<br>Implants…………… **C**<br>Female sterilization…………… **D**<br>Patch…………… **E**<br>IUD…………… **F**<br>Rhythm (Periodic Abstinence) …………… **G**<br>Condoms…………… **H**<br>Vasectomy…………… **I**<br>Emergency contraception…………… **J**<br>Withdrawal…………… **K**<br>Other (specify)________________ **X**<br>________________<br>________________
**[If provider selected all contraceptive methods in Q. 309, skip to Module 4]**		
310	Why do you offer these methods and not others?<br><br>**[Do not read list. Multiple responses are allowed]**	Those are the ones we have in stock…………. A<br>Provider preferences based on training………. B<br>Provider experience of what has worked well for other women……………….. C<br>Provider preferences based on woman's charcteristics……………. D<br>Client preferences…………… E<br>Not familiar enough with other methods………. F<br>Religious reasons…………… G<br>Funder's restriction…………… H<br>Other (specify): ________________ X

Module IV: General

Now we would like ask you some general questions about your opinion about the Kenyan abortion law, your opinion on barriers to the provision of PAC, as well as how services can be improved.

401	Do abortion complications form a significant portion of the cases that are seen/ treated in this facility?	Yes…………………… 1<br>No…………………… 2 **[Go to 403]**
402	How do you consider the treatment of abortion complications to be a burden for your facility?<br>**[Circle all that apply. DO NOT Prompt]**	Number of patients……………………………………… **A**<br>Equipment……………………………………………… **B**<br>Supplies………………………………………………… **C**<br>Space/ Infrastructure………………………………… **D**<br>Human resources/ Qualified personnel……………… **E**<br>Client's inability to pay……………………………… **F**<br>No burden…………………………………………….. **G**<br>Other (Specify) ______________________ **X**
403	Up until now we have been talking of both induced and spontaneous abortions together. Now, I'd like you to think specifically on the causes of 2nd trimester spontaneous abortions only. What are the causes of 2nd trimester spontaneous abortions among women who seek care in this facility? **[Do not Prompt]**<br>Out of 100 2nd trimester spontaneous abortion cases that present in this facility, how many spontaneous abortions would you say are due to the causes you have mentioned in the 2nd trimester?	See table below
404	In your opinion, how could treatment for abortion complications be improved at this facility?	______________________<br>______________________<br>______________________<br>______________________<br>______________________
405	Currently, the law only permits abortion to protect the life and health of the woman.<br><br>**Do you think** the abortion law in Kenya should be changed?	Yes……………………………… 1<br>No……………………………….. 2 **[Instructions preceeding Q407]**<br>Don't know /No opinion………. 3
406	What changes do you want to see in the law?<br><br>**Prompt: Multiple responses allowed**	**Abortion should be provided:**<br><br>On demand (if the woman requests for it)………………… **A**<br>If the woman's health is at risk……………………………… **B**<br>If the girl is still in school……………………………………… **C**<br>Unmarried girl/woman ………………………………………… **D**<br>If the girl/woman cannot care for the child………………… **E**<br>If the mother is HIV+…………………………………………… **F**<br>Pregnancies after incest……………………………………… **G**<br>Pregnancies after rape………………………………………… **H**<br>Contraceptive failure……………………………………….. **I**<br>Abortion should not ever be provided……………………… **J**<br>Other (Specify) ______________________ **X**<br>______________________

Question 403 responses:

	TICK	%
Malnutrition…………………………		
HIV……………………………………		
Malaria………………………………		
Congenital malformation……………		
Maternal complications……………		
Domestic Violence…………………		
Sexually transmitted infections (excluding HIV)……………………		
Urinary tract infections………..		
Trauma/heavy work……………		
Does not see spontaneous abortions/Don't know……………		
Other (Specify)______________		
______________________		
Total ………………………………		100%

407	Do you think the Health Management Information System (HMIS) at this facility under counts post-abortion care cases?	Yes…………………………………… 1<br>No…………………………………… 2

END TIME: ___ ___ hr ___ ___ min

INTERVIEWER'S COMMENTS____

____

____

____

____

____

ASK TO TALK TO THE HMIS OFFICER. REQUEST TO GET THESE RECORDS FOR THE YEAR 2011

	JAN	FEB	MAR	APR	MAY	JUN	JUL	AUG	SEP	OCT	NOV	DEC
NO. OF POST ABOTION CARE												

If can't access HMIS record, explain why? ____

____

____
