# Supplementary material for: Spatial and socio-economic correlates of effective contraception among women seeking post-abortion care in healthcare facilities in Kenya
Source: PLoS One. 2019 Mar 27;14(3):e0214049. doi: 10.1371/journal.pone.0214049 (PMC6436713; doi:10.1371/journal.pone.0214049)
Supplement: S1 File — Authority to use data and questionnaires used to collect the data used in this study. (ZIP) [file pone.0214049.s001.zip › Supporting information/S2 PONE-D-17-43970_Health Professionals Survey Questionnaire.pdf]

Ministry of Medical Services (MOMS) | African Population & Health Research Center (APHRC) | Division of Reproductive Health, Ministry of Public Health & Sanitation

STUDY OF ABORTION AND WOMEN'S HEALTH IN KENYA 2012

SURVEY OF HEALTH PROFESSIONALS IN KENYA

KEMRI's Ethical Review Committee protocol number 320

IDENTIFICATION

**K1**	PROVINCE……………………………………………….	|__|__|__|__|__|__|__|__|
**K2**	DISTRICT………………………………………………..	|__|__|__|__|__|__|__|__|
**K3**	COUNTY……………………………………………….	|__|__|__|__|__|__|__|__|
**K4**	RESPONDENT'S IDENTIFICATION NUMBER……….	|__|__|__|__|__|__|__|__|
**K5**	WHERE RESPONDENT WORKS………………….	______________________
**K6**	DATE OF INTERVIEW……	|__|__| Day |__|__| Month **2012** Year
**K7**	CURRENT LOCATION OF WORK<br>***[Interviewer: If respondent works in both rural and urban areas, circle the category corresponding to the area where he/she contributes the most time.]***	1 Rural area<br>2 Urban area
**K8**	START TIME |__|__| **h** |__|__| **mn**	END TIME |__|__| **h** |__|__| **mn**
**K9**	INTERVIEWER CODE…………………….....	|__|__|__|__|__|__|__|__|

For interviewer to fill out at the end of the interview:

**INTERVIEWER COMMENTARY ON RESPONDENT'S KNOWLEDGE OF RURAL AREAS**		**INTERVIEW RESULT**	
KNOWS VERY WELL………………………………………	**1**	COMPLETE…………………………………………………	**1**
KNOWS MODERATELY WELL……………………………	**2**	REFUSED……………………………………………………	**2**
KNOWS PASSABLY WELL………………………………	**3**	INCOMPLETE………………………………………………	**3**
DOES NOT KNOW………………………………………	**4**	NOT AVAILABLE FOR INTERVIEW………………………	**4**

COMMENTS:……………………………………………………………………………………...……...……..
………
………
………
………
………
………

Module I: Basic Information

	Questions and Instructions	Responses, Codes and Filters
101	Sex of respondent<br>***[Interviewer: Circle the cell that applies to the respondent.]***	1 Male<br>2 Female
102	How old are you?	☐☐ Years
103	Which of the following categories describes your primary profession?<br>***[Interviewer: If more than one applies, circle the category that accounts for the greatest proportion of the respondent's time]***	1 Researcher<br>2 OB-GYN specialist<br>3 Nurse<br>4 Midwife<br>5 Clinical Officer<br>6 Medical Officer/GP<br>7 Health Administrative Officer (e.g in MoH)<br>8 Lawyer<br>9 Counselor/social work<br>10 Advocacy (specify) ________________<br>11 Other (specify) ___________________
104	How long have you been working in this field?	☐☐ Years
105	In which sector do you work primarily?<br>***[Interviewer: If the respondent works in more than one sector, tick the category corresponding to the sector where he/she contributes the most time. If the respondent works equally in both the private and the public sector, they can fill that in under "Other".]***	1 Public sector (government)<br>2 Private for profit sector<br>3 Private-not-profit (Non-governmental organization/CBO)<br>4 Other (specify) ____________________
106	Do you have experience working in rural areas for six months or more in the last five years?	1 Yes<br>2 No<br>List the names of the rural areas where you have worked for six months or more in the last five years<br>________________________________<br>________________________________<br>________________________________

Module II: Service Provision

There is so much talk on abortion everywhere in the province. In the next section we are going to talk about abortion. When we talk about woman, we mean any female who can become pregnant. We would like to have your opinion about abortion care and associated problems in Kenya.

201 Please identify the different situations in which you have encountered the issue of abortion.

[Interviewer : Please tick all that apply, but do not suggest the answers]

1	Personally, in a public health center
2	Personally, in a private health center
3	Personally, in a non-medical framework (research, policy-making, counseling, advocacy, law, etc.)
4	Through colleagues in any of the above settings
5	Other (specify) ______________________________

202 As far as you are aware, what methods are used in rural areas of Kenya to induce abortion?

[Interviewer: Please read out each type of method for rural areas, tick the appropriate response and then repeat the same questions for urban areas. Please tick all that apply from the list below, regardless of the type of practitioner who may use the method.]

How about in urban areas?

Type of Method	1. Rural Areas			2. Urban Areas		
	1. Yes	2. No	3. Don't Know	1. Yes	2. No	3. Don't Know
**a. Dilation and Evacuation (D&E)**						
**b. Dilation and curettage (D&C)**						
**c. Manual vacuum aspiration (MVA)**						
**d. Electric vacuum aspiration (EVA)**						
**e. Medication abortion (e.g. Cytotec/misoprostol)**						
**f. Oral introduction of drugs, solutions or other substances (e.g. through the mouth)**						
i. Hormonal drugs (e.g. Contraceptive pills)						
ii. Herbs/ Teas/ Solutions						
iii. Caustic agents (e.g. Washing detergent)						
iv. Overdose of pharmaceuticals (e.g. Quinine, SP 3 tablets)						
v. Other (Specify) _________________________						
**g. Injectables specify______________________________**						
**h. Cervical/ Vaginal introduction of drugs, solutions or other materials**						
i. Hormonal drugs (e.g. Contraceptives)						
ii. Herbs/Teas/ Solutions (Using form of insertion)						
iii. Catheter						
iv.Piercing objects (e.g. sticks, wires, knitting needles)						
v. Other (specify) _________________________						
**i. Heavy massage/physical exertion, physical blows, jumping, falling, marching**						
**j. Other means (Specify any additional method/s not listed above)________________________________**						

203 What do you think is the MOST common method used by:

[Interviewer:use the letters in Q.202]

	Rural areas	Urban Areas
TBA/Tradidional healer	___________	___________
Clinical officer/ Nurse/ Midwife	___________	___________
Doctor	___________	___________
Woman herself (self-induction)	___________	___________

The following questions are asked separately about women who live in rural and urban areas. Each one asks you to consider two broad income groups – the poor and the relatively well-off (non-poor). Looking at this province as a whole and bearing in mind the differences in this province, I want you to give us your opinion on the following.

[Interviewer: You can mention that there are not exact definitions for "poor" and "non-poor," but by "poor" we mean women with lower income levels/cash incomes.]

RURAL AREAS

204 I will mention the main types of people who perform induced abortions in Kenya. Considering first **rural areas**, indicate whether, in your opinion, each type of provider is used rarely, sometimes or commonly by poor rural women seeking abortion.

[Interviewer: Please read each type of provider and circle the respondent's answers for poor rural women. Mark all the respondent's answers relating to poor rural women, then ask the next question.]

Now indicate whether, in your opinion, each type of provider is used rarely, sometimes or commonly by **non-poor rural** women.

**Type of Provider**<br>**Read each type**	N=Never/Rarely<br>S=Sometimes<br>C=Commonly	**1. Poor rural women**			**2. Non-poor rural women**		
		N/R	S	C	N/R	S	C
a. TBA/traditional healer		1	2	3	1	2	3
b. Clinical officer		1	2	3	1	2	3
c. Nurse		1	2	3	1	2	3
d.Trained Midwife		1	2	3	1	2	3
e. Doctor		1	2	3	1	2	3
f. Pharmacist/Chemist		1	2	3	1	2	3
g. Woman - self-induced		1	2	3	1	2	3
h. Other *(specify)* : ______________________________		1	2	3	1	2	3

205 How much do you think women in rural **areas** pay for first trimester abortions, according to the type of provider they use?

[Interviewer: Please check with Q 204 and request an amount or price range for only those providers who were rated as used commonly or used sometimes].

**Type of Provider**	**Amount Paid (KES)**	
	**1. Minimum**	**2. Maximum**
a. TBA/traditional healers		
b. Clinical officer		
c. Nurse		
d. Trained midwife		
e. Doctor		
f. Pharmacist/Chemist		
g. Woman herself		
h. Other (specify) ________________		

206 In your opinion, what percent of all induced abortions in **rural areas** do you think are being performed by each type of provider for **poor** women? Give an approximate percentage (all providers sum to 100%).

Now let's turn to non-poor women who live in rural **areas**.

[Interviewer: Confirm that all providers sum to 100%. If they do not, probe for a correction, and adjust the answers below.]

**Type of Provider**	**1. Rural Poor**	**2. Rural Non-Poor**
a. TBA/traditional healers		
b. Clinical officer		
c. Nurse		
d. Trained midwife		
e. Doctor		
f. Pharmacist/Chemist		
g. Woman- Self induced		
**Total**	**100%**	**100%**

URBAN AREAS	
207	I will mention the main types of people who perform induced abortions in Kenya. Considering first urban **areas**, indicate whether, in your opinion, each type of provider is used rarely, sometimes or commonly by poor **urban** women seeking abortion.<br><br>**[Interviewer: Please read each type of provider and circle the respondent's answers for poor urban women. Mark all the respondent's answers relating to poor urban women, then ask the next question.]**<br><br>Now indicate whether, in your opinion, each type of provider is used rarely, sometimes or commonly by **non-poor urban** women.

Type of Provider<br>Read each type	R=Rarely/Never<br>S=Sometimes<br>C=Commonly	1. Poor urban women			2. Non-poor urban women		
		N/R	S	C	N/R	S	C
a. TBA/traditional healer		1	2	3	1	2	3
b. Clinical officer		1	2	3	1	2	3
c. Nurse		1	2	3	1	2	3
d. Trained Midwife		1	2	3	1	2	3
e.Doctor		1	2	3	1	2	3
f. Pharmacist/Chemist		1	2	3	1	2	3
g. Woman - self-induced herself		1	2	3	1	2	3
h. Other *(specify)* : ______________________________		1	2	3	1	2	3

208 How much do you think women in **urban areas** pay for first trimester abortions, according to the type of provider they use?

[Interviewer: Please check with Q 207 and request an amount or price range for only those providers who were rated as used commonly or used sometimes. If they do not, probe for a correction, and adjust answers below]

Type of Provider	Amount Paid (KES)	
	**1. Minimum**	**2. Maximum**
a. TBA/traditional healers		
b. Clinical officer		
c. Nurse		
d. Trained midwife		
e. Doctor		
f. Pharmacist/chemist		
g. Woman - self induced herself		
h. Other (specify) ________________		

209 In your opinion, what percent of all induced abortions in urban **areas** do you think are being performed by each type of provider for **poor** women? Give an approximate percentage (all providers sum to 100%).

[Interviewer: Confirm that all providers sum to 100%. If they do not, probe for a correction, and adjust the answers below. Allow the respondent to fully answer the question for poor women living in urban areas, then ask the following question.]

Now let's turn to **non-poor** women who live in urban **areas.**

Type of Provider	**1. Urban Poor**	**2.Urban Non-Poor**
a. TBA/traditional healers		
b. Clinical officer		
c. Nurse		
d. Trained midwife		
e. Doctor		
f. Pharmacist/Chemist		
g. Woman- Self induced		
**Total**	**100%**	**100%**

	Not all abortions that happen in this province are unsafe. There could be situations under which a woman is able to obtain an abortion that does not result in any complications for the woman. Now I am going to ask you a few questions about how often you think abortions don't result in any complications for the four sub-groups we've been discussing above.	
210	Thinking now of (i) rural **poor** women in the province, what percent of abortions would you say are without complications?<br><br>**Now thinking about (ii) rural nonpoor women....**	**1. Rural**: i) Poor ___ / ii) Nonpoor ___
211	Thinking now of (i) urban **poor** women in the province, what percent of abortions would you say are without complications?<br><br>**Now thinking about (ii) urban nonpoor women....**	**2. Urban**: i) Poor ___ / ii) Nonpoor ___
212	Thinking now of (i) rural poor women in the province, what proportion of abortions by a trained provider using a safe abortion technique are without health complications?<br><br>Thinking now of (ii) rural nonpoor women in the province,	**1. Rural**: i) Poor ___ / ii) Nonpoor ___
213	Thinking now of (i) urban poor women in the province, what proportion of abortions by a trained provider using a safe abortion technique are without health complications?<br><br>Thinking now of (ii) urban non-poor women in the province,	**2. Urban**: i) Poor ___ / ii) Nonpoor ___
214	Thinking now of rural women in your region, for every 10 abortions that are done, how many would you say are repeat abortions?<br><br>Now thinking about urban women....	**1. Rural** ___ / **2. Urban** ___
215	In your opinion, what are the reasons for repeat abortion among rural women?<br><br>In your opinion, what are the reasons for repeat abortion among urban women?<br><br>**[Interviewer: Do not prompt. Tick all that apply]**	See list below

	Reason	Rural	Urban
A	Poverty		
B	Lack of access to contraceptives		
C	Lack of negotiation power in sex		
D	Immorality		
E	Contraceptive failure		
F	Objection to contraceptive use		
G	Non-adherence to contraceptives		
H	Lack of information		
I	Others (specify)_____________________________		

Module III: Abortion Complications

Now, I would like to talk to you about abortion complications. As you know, complications that result from induced abortion range in severity. When we speak of abortion complications, we are referring to those consequences ***that are severe enough to need treatment in a health facility,*** *whether or not the woman actually obtains health care.*

301 Considering rural areas, please indicate which forms of short term health complications apart from death, result commonly, sometimes or never from induced abortion in your province.

Now considering urban areas....

[Interviewer: Please read each type of complication and circle the respondents answers for each type of complication]

N=Never; S=Sometimes; C= Commonly

		Rural areas			Urban Areas		
		N/R	S	C	N/R	S	C
A	Incomplete abortion	1	2	3	1	2	3
B	Excessive loss of blood	1	2	3	1	2	3
C	Infection of the uterus and/or surrounding areas	1	2	3	1	2	3
D	Sepsis or septic shock	1	2	3	1	2	3
E	Damage to gut (intestines)	1	2	3	1	2	3
F	Damage to vagina and cervix	1	2	3	1	2	3
G	Damage to uterus (e.g. Perforation)	1	2	3	1	2	3
H	Pain in lower abdomen	1	2	3	1	2	3
I	Other	1	2	3	1	2	3

(specify, rural) ____________________________

(specify urban) ____________________________

Now I'm going to ask you some questions about the experiences of women who live in rural areas. I want you to think about women in this province when you answer the questions that follow.

RURAL AREAS

302 **RURAL AREAS**

Think about **poor women** in **rural areas**: Out of ten **poor rural women** who have an abortion performed by each type of provider that I will mention, how many would experience a medical complication that should receive medical treatment?

What would the number be for **non-poor women** in **rural areas?**

[Interviewer: Ask for each type of provider separately; insert a number in each column, even though it might be "0."]

Rural Areas	1. TBA/Traditional healer	2. Clinical Officer	3. Nurse	4. Trained midwife	5. Doctor	6. Pharmacist/ Chemist	7. Woman herself
**a. Poor:** Number out of 10 women with abortion complications							
**a. Non-Poor:** Number out of 10 women with abortion complications							

303 Think about **poor women** in **rural areas**: out of 10 poor urban women who experience **an abortion complication** due to an induced abortion, how many do you think would be treated in a health facility?

[Interviewer: Allow the respondent to fully answer this question and then ask the following question.]

What would the number be for **non-poor women** living in **rural** [illegible]

Number out of 10 treated in health facility

1	Rural poor	☐☐
2	Rural non-poor	☐☐

304 Where can women who live in **rural areas** go to be treated for abortion complications that result from induced abortion? Please indicate whether, in your opinion, each type of provider is used (1) Never/Rarely, (2) Sometimes, (3) Commonly by **poor rural** women?

Now let's turn to **non-poor** (relatively well-off) women living in **rural** areas.

[Interviewer: Please read out each type of provider and then tick the appropriate section (neve/rarely, sometimes, commonly) mentioned for rural poor, rural non-poor, respectively.]

[Interviewer: Please read out each type of provider and then mark the appropriate number (1, 2, or 3) for rural poor and rural non-poor.]

1= Never/Rarely
2= Sometimes
3= Commonly

Type of Provider	Rural areas			Urban Areas		
	N/R	S	C	N/R	S	C
a. TBA/Traditional healer	1	2	3	1	2	3
b. Clinical officer	1	2	3	1	2	3
c. Nurse	1	2	3	1	2	3
d. Trained midwife	1	2	3	1	2	3
e. Doctor	1	2	3	1	2	3
f. Pharmacist/Chemist	1	2	3	1	2	3
g. Woman-Self-induced	1	2	3	1	2	3
h. Other (specify) _______________	1	2	3	1	2	3

Now I'm going to ask you some questions about the experiences of women who live in urban areas. I want you to think about women in this province when you answer the questions that follow.

URBAN AREAS

305 **URBAN AREAS**

Think about **poor women** in **urban areas**: Out of ten poor urban women who have an abortion performed by each type of provider that I will mention, how many would experience a medical complication that should receive medical treatment?

What would the number be for **non-poor women** in **urban areas?**

[Interviewer: Ask for each type of provider separately; insert a number in each column, even though it might be "0."]

Urban Areas	1. TBA/Tradition-al healer	2. Clinical Officer	3. Nurse	4. Trained midwife	5. Doctor	6. Pharmacist/ Chemist	7. Woman herself
**a. Poor:** Number out of 10 women with abortion complications							
**a. Non-Poor:** Number out of 10 women with abortion complications							

306 Think about **poor women** in **urban areas**: Out of 10 poor urban women who experience **an abortion complication** due to an induced abortion, how many do you think would be treated in a health facility?

[Interviewer: Allow the respondent to fully answer this question and then ask the following question.]

What would the number be for **non-poor women** living in **urban areas?**

Number out of 10 treated in health facility

1	Urban poor	☐☐
2	Urban non-poor	☐☐

307 Where can women who live in **urban areas** go to be treated for abortion complications that result from induced abortion? Please indicate whether, in your opinion, each type of provider is used (1) Never/Rarely, (2) Sometimes , or (3) Commonly by **poor rural** women?

Now let's turn to **nonpoor** (relatively well-off) women living in **urban** areas.

[Interviewer: Please read out each type of provider and then tick the appropriate section (never/rarely, sometimes, commonly) mentioned for urban poor, urban non-poor, respectively.]

[Interviewer: Please read out each type of provider and then mark the appropriate number (1, 2, or 3) for urban poor and urban non-poor.]

1= Never/Rarely
2= Sometimes
3= Commonly

**Type of Provider**	Rural areas			Urban Areas		
	N/R	S	C	N/R	S	C
a. TBA/Traditional healer	1	2	3	1	2	3
b. Clinical officer	1	2	3	1	2	3
c. Nurse	1	2	3	1	2	3
d. Trained midwife	1	2	3	1	2	3
e. Doctor	1	2	3	1	2	3
f. Pharmacist/Chemist	1	2	3	1	2	3
g. Woman-Self-induced	1	2	3	1	2	3
h. Other (specify) _______________	1	2	3	1	2	3

Now I would like to talk to you about women with late term spontaneous abortions.

308 In your opinion, among 10 women who have spontaneous abortion in the first trimester, how many are likely to seek care from a skilled health provider?

309 In your opinion, among 10 women who have spontaneous abortion in the second trimester, how many are likely to seek care from a skilled health provider?

	Rural	Urban
First Trimester		
Second Trimester		

310 In your opinion, what are the barriers for use of postabortion care services for women who need care by women? Please state if it is never/rarely, sometimes or common.

[Interviewer: Please indicate (1) Never/Rarely, (2) Sometimes, or (3) Commonly]

Barriers for use of PAC services

		N/R	S	C
1	Cost to woman			
2	Distance/ transportation	1	2	3
3	Inadequate training of providers	1	2	3
4	Inadequate space/equipment at facility	1	2	3
5	Under-staffing at facility	1	2	3
6	Hostile/unfriendly provider attitudes	1	2	3
7	Lack of patient information on services	1	2	3
8	Husband/family objections	1	2	3
9	Fear/stigma	1	2	3
10	Delay in identifying danger signs	1	2	3
96	Other (specify):_______________	1	2	3

311	How often do you think the following barriers prevent women from using postabortion care services in health facilities? Please state if it is never/rarely, sometimes or common.<br><br>**[Interviewer: Please indicate (1) Never/Rarely, (2) Sometimes, or (3) Commonly]**	

		N/R	S	C
1	Reduce cost	N/R	S	C
2	Increase services in rural areas	1	2	3
3	Male involvement in RH issues	1	2	3
4	Improve provider training	1	2	3
5	Provide more or better equipment	1	2	3
6	Increase staffing	1	2	3
7	Retrain providers to change attitudes	1	2	3
8	Provide information to women on services	1	2	3
9	Involvement of CHWs	1	2	3
98	Other (specify):_________________	1	2	3

312	The preceeding sections included questions that required you to give your opinion on concepts that are not easily measured. On a scale of 1 to 10, with 1 being "not at all sure" and 10 being "very sure", what is your degree of certainty that the answers you've given reflect the real situation encountered in your province?	10	Very sure
		9	
		8	
		7	
		6	
		5	
		4	
		3	
		2	
		1	Not at all sure

Module IV: Postabortion Counselling

401	As you know, some women become pregnant while they are using contraceptive methods.<br><br>In your opinion, which methods were women with unintended pregnancies using at the time of conception?<br><br>**[Interviewer: Multiple responses are allowed. Do not prompt]**<br><br>**Probe for other methods.**<br>(Are there other methods? What else...)	A Pills<br>B Injectables<br>C Implants<br>D IUD<br>E Male condom<br>F Female condom<br>G Diaphram<br>H Tubal ligation (Female sterilization)<br>I Vasectomy (Male sterilization)<br>J Withdrawal<br>K Rhythm method (Periodic abstinence)<br>L Spermicides<br>M Emergency contraception<br>N Abstinence<br>O No method<br>P Lactational amenorrhea<br>X Other (specify):__________________
402	Do you think providers in this province are aware of post abortion contraceptive couselling guidelines?	☐ Most of them<br>☐ Few of them<br>☐ None of them
403	Do you think providers of PAC services in this province offer counselling services?	☐ Most of them<br>☐ Few of them<br>☐ None of them
404	Do you think providers should offer postabortion contraceptive counselling services to all, some, or no abortion patients?	1 All<br>2 Some<br>3 None
405	Do you think that all abortion patients should be offered postabortion contraceptive counseling while they are still at the health facility?	☐ Yes<br>☐ No
406	Do you think that all abortion patients should be offered a contraceptive method while they are still at the health facility?	☐ Yes<br>☐ No<br>☐ Sometimes

407	In your opinion, which methods are most appropriate for post abortion care patients?<br><br>**[Interviewer: Multiple responses are allowed. Do not prompt]**<br><br>**Probe for other methods.**<br>(Are there other methods? What else...)	A Pills<br>B Injectables<br>C Implants<br>D IUD<br>E Male condom<br>F Female condom<br>G Diaphram<br>H Tubal ligation (Female sterilization)<br>I Vasectomy (Male sterilization)<br>J Withdrawal<br>K Rhythm method (Periodic abstinence)<br>L Spermicides<br>M Emergency contraception<br>N Abstinence<br>O No method<br>X Other (specify):_________________

Module V: Abortion and the Constitution	
501	What does the Kenyan Law say about abortion?
502	What is the difference between the **old** and the **new** constitution on abortion?
503	What do you think of these changes?
504	So far, how has the new constitution influenced abortion provision in Kenya?
505	What do you think would be the best way of using the new constitution to prevent unsafe abortion in Kenya?

506	Under what circumstances do you think that abortion should be legal?<br><br>**[Interviewer : Multiple responses are allowed. Do not prompt.]**		
		a. If woman's health is at risk	A
		b. For social or economic reasons (for e.g. if the girl is still in school, unmarried or cannot care for a child)	B
		c. On demand	C
		d. Contraceptive failure	D
		e. Rape	E
		f. Incest	F
		g. Other	G
		(specify) : ________________________________	
507	Please indicate any suggestions / recommendations that you think could be used in Kenya to reduce the number of unsafe abortions.<br><br>**[Interviewer : Multiple responses are allowed. Do not prompt.]**		
		a. Publicize the health risk associated with unsafe abortion (awarene	A
		b. Provide counseling services after delivery and after abortion	B
		c. Increase availability of family planning services / Improve access to effective contraception	C
		d. Involve men	D
		e. Educate women on how to negotiate safer sex	E
		f. More liberal laws on abortion	F
		g. Make abortion illegal	
		h. Other (*specify*) : ________________________________	G
508	What changes would you like to see in PAC service delivery ?<br>____<br>____<br>____<br>____<br>____<br>____		
	Are there any other comments you would like to give us?<br>____<br>____<br>____<br>____<br>____<br>____<br>____<br>____<br>____<br>____<br>***END: Thank you for your cooperation***		
**Turn back to the front page to finish filling out the cover page**			
